# Positional Correlation Length-Induced Morphological Transformation of Interpolyelectrolyte Complexes (IPECs) Made of Polysaccharides: The Role of Molar Charge Ratio

**DOI:** 10.1021/acs.macromol.5c01621

**Published:** 2025-09-26

**Authors:** Avik Das, Sylvain Prévost, Michael Gradzielski

**Affiliations:** † Stranski-Laboratorium für Physikalische und Theoretische Chemie, Institut für Chemie, 26524 Technische Universität Berlin, D-10623 Berlin, Germany; ‡ Large Scale Structures, 56053 Institut Laue-Langevin − The European Neutron Source, 71, Avenue des Martyrs, 38042 Grenoble, France

## Abstract

By combination of oppositely charged polyelectrolytes, one can form interpolyelectrolyte complexes (IPECs) that may serve, for example, as water-based delivery systems. Such macromolecular IPECs may exhibit broad size and shape distributions; thus, quantitative structural analysis is often prone to misinterpretation. By means of small-angle neutron scattering (SANS), we present a systematic analysis of the internal macromolecular structures of interpolyelectrolyte complexes (IPECs) in a quantitative fashion. As a model system, we study polyelectrolyte mixtures of anionic biosourced sodium carboxymethyl cellulose (NaCMC) and cationic synthetic poly­(diallyldimethylammonium chloride) (PDADMAC). By integrating model-independent and model-dependent approaches, we reveal a morphological transformation from larger globular aggregates to smaller, multimodal lumpy structures with increasing molar charge ratio, i.e., upon approaching charge equimolarity. At the same time, the aggregates undergo significant compaction, driven by local structuring and a reduction in the characteristic correlation length of charge distribution, and they become increasingly anisometric. Additionally, the complexes retain a substantial amount of solvation water, which is gradually released as the charge ratio approaches equimolarity.

## Graphical abstract

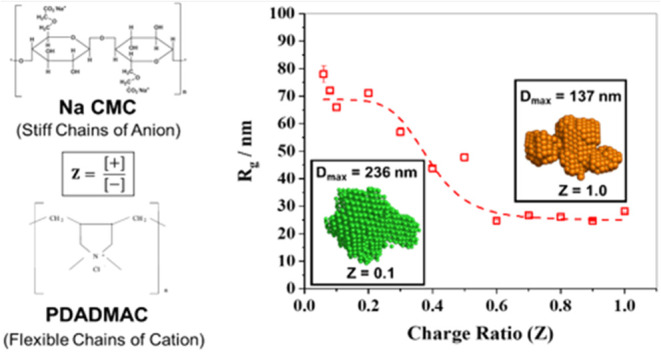

## 1 Introduction

Interpolyelectrolyte complexes (IPECs) are macromolecular structures that spontaneously emerge due to associative aggregation when solutions of oppositely charged polyelectrolytes (PEs) are combined.^1–3^ Formation of IPECs is a phenomenon mainly associated with the entropy-driven release of counterions and solvent molecules in solution.^4,5^ IPECs may exist in the form of a homogeneous solution of colloidal particles dispersed in a liquid phase or undergo phase separation, leading to either precipitation (liquid–solid phase separation) or coacervate formation (liquid–liquid phase separation).^6–8^ Dispersed complexes are typically formed when a significant excess charge exists on one of the PEs, whereas phase separation occurs in the vicinity of charge neutralization. IPECs have been proven to be versatile materials with diverse applications, including gene delivery,^9,10^ underwater adhesion,^11,12^ coatings,^13,14^ and nanofiltration-based separations.^15,16^ Designing IPEC-based materials for tailor-made applications requires a comprehensive understanding of the role of polyelectrolyte architecture in controlling the complex internal macromolecular structure of IPECs. Detailed knowledge about the formation of complex macromolecular structures and the underlying driving forces can enable the fine-tuning of these structures to use them as drug carriers, bioadhesives, and coating materials.

The internal structure of IPECs is mainly governed by the electrostatic interactions between charge correlation volumes, called “blobs”.^17–19^ There exists an intricate balance between the Coulomb attraction and Coulomb repulsion as well as the degrees of freedom offered by the polymeric chains, leading to a spatial distribution of electrostatic blobs of polycations and polyanions. The internal structure of IPECs can be understood by using the positional correlation of electrostatic blobs. Koberstein et al.^20^ suggested that there exists a long-range correlation among randomly distributed inhomogeneities with a correlation length much greater than the radius of gyration of individual polyelectrolytes dissolved in the solution. Further, recent work by Fang et al.^21^ provided experimental evidence of the positional correlation between electrostatic domains in liquid polyelectrolyte complexes by means of small-angle neutron scattering (SANS). In the electrostatic blob model, polyelectrolyte chains restructure themselves into small domains with locally correlated charges. These domains serve as fundamental structural units, and their size and spatial arrangement are primarily dictated by charge stoichiometry, ionic strength, polyelectrolyte rigidity (intrinsic and electrostatic persistence length), counterion condensation, and solvation effects. However, a more comprehensive correlation between the spatial distribution of electrostatic blobs and the overall morphology of macromolecular aggregates is still lacking. Understanding this correlation can provide valuable insights into the morphological evolution of the macromolecular aggregate and enable fine-tuning of the functional properties of polyelectrolyte complexes.

Over the past 10 years, various polysaccharide-based polyelectrolytes, such as alginate, chitosan, cellulose, and its derivatives,^22,23^ have been used as functional components to prepare macromolecular complexes for various applications, including food,^24^ medicine,^25^ and the environment.^15^ Among various polysaccharides, sodium carboxymethyl cellulose (NaCMC), an anionic water-soluble polysaccharide derived from cellulose, has the ability to form macromolecular complexes with the potential to serve as pH-responsive drug delivery and controlled release systems.^26–28^ The biopolyelectrolyte sodium carboxymethyl cellulose (NaCMC) is based on a polysaccharide backbone and generally exhibits excellent biocompatibility, biodegradability, and nontoxicity.^29,30^ Due to the structure of the polysaccharide backbone with its large number of OH-groups, they and their complexes have a natural tendency to bind larger amounts of water. This distinguishes them from typical synthetic polyelectrolytes like poly­(diallyldimethylammonium chloride) (PDADMAC), which is positively charged in aqueous solutions owing to quaternary ammonium groups. It is used in the mining industry,^31^ paper manufacturing,^32^ and water treatment.^15^ In addition, PDADMAC has frequently been employed in the formation of IPECs, for instance, with poly­(styrenesulfonate) (PSS)^33^ or modified poly­(acrylic acid) (PAA).^34^ In addition, the precipitates it forms with NaCMC can serve as model surfaces for paper fibers.^35^

In our work, we employed a mixture of synthetic and biopolyelectrolytes, which are rather asymmetric in terms of their backbone structures and tendency to bind water, in addition to ionic solvation. An intriguing aspect of this system is how the stiffer chains of the polysaccharides influence the complexation behavior. NaCMC has a reported persistence length of approximately 10–20 nm,^36^ which is significantly higher than the ∼2–3 nm persistence length of the synthetic polyelectrolyte PDADMAC.^37^ This pronounced asymmetry in chain flexibility introduces a complex interplay between the enthalpic and entropic contributions during complexation. The rigid nature of the polysaccharide backbone imposes spatial constraints on chain mobility, which may hinder interpenetration and affect the formation of compact complexes. Additionally, the hydration shell around the carboxymethyl groups in NaCMC is more structured and extensive compared to that of PDADMAC,^38,39^ potentially influencing the thermodynamics of complex formation. These disparities in chain flexibility and hydration may result in complex internal structures. The nature of water–polymer interactions also raises questions about the extent to which bound water mediates or interferes with electrostatic interactions between oppositely charged groups.^39^ As IPECs form, partial dehydration of the polyelectrolytes is often required to facilitate ion pairing, and this step may be more energetically costly for NaCMC. Thus, this system provides an excellent model to elucidate how molecular conformation and water affinity together govern complex formation under different stoichiometric conditions.

Building upon the fundamental principles of IPEC formation, the present work delves into the intricate structural characteristics of complexes formed between anionic polysaccharide sodium carboxymethyl cellulose (NaCMC) and the cationic synthetic polymer poly­(diallyldimethylammonium chloride) (PDADMAC). By systematically varying the molar charge ratio of PDADMAC to NaCMC, we aimed to elucidate the structural evolution of the resulting IPECs across a range of mixing conditions, particularly when approaching charge neutrality. We maintained a fixed NaCMC concentration across all samples to provide a consistent anionic framework, allowing us to study how the incremental addition of PDADMAC influences the progression of IPEC formation. This framework is essential to observe sequential structural transitions due to the increasing polycation availability. However, it is noteworthy that with this experimental configuration, the total amount of polycation and polyanion, and the ionic strength, also increase, potentially influencing the complexation behavior. While decoupling these variables would be ideal for isolating electrostatic effects, the current study aims to present the integrated response of the system as a function of the mixing ratio of polycation to polyanion. To achieve this, we primarily employed small-angle neutron scattering (SANS), a powerful technique sensitive to structural features at the nanoscale, complemented by static (SLS) and dynamic light scattering (DLS), as well as ζ-potential measurements to gain structural insights into the self-assembled aggregates in solution. Our objective was to establish a quantitative correlation between the internal architecture and overall morphology of IPECs as a function of their stoichiometric composition. Through both model-independent and model-dependent real-space analyses of the scattering data, we could extract a coherent and comprehensive understanding of the structural transitions and organizational principles governing these macromolecular assemblies as the mixing ratio approached equimolarity.

## 2 Experimental Methodology

### 2.1 Materials

Poly­(diallyldimethylammonium chloride) (PDADMAC) (*M*_w_: ∼400–500 kDa) of 20 wt % solution in H_2_O and sodium carboxymethyl cellulose (NaCMC) (average *M*_w_: ∼250 kDa, degree of substitution, 0.7) were purchased from Sigma-Aldrich. D_2_O was used as a solvent for preparing samples for neutron scattering characterization; otherwise, H_2_O was used. Milli-Q water was produced by a Millipore filtering system. D_2_O was obtained from Eurisotop (99.5% isotopic purity, Gif-sur-Yvette, France). D_2_O was filtered through a PTFE syringe filter with a pore size of 0.45 μm to eliminate any potential dust particles.

### 2.2 Preparation of Interpolyelectrolyte Complexes (IPECs)

To begin with, a stock solution of the polyanion (NaCMC) at a concentration of 15 g/L was prepared by dissolving NaCMC powder in either H_2_O or D_2_O under strong stirring conditions. Subsequently, a stock solution of the polycation (PDADMAC) at a concentration of 10 g/L was prepared by appropriately diluting the supplied solution. The charge ratio, denoted as *Z*, was defined as the ratio of the molar charge per monomer of polycation ([+]) to that of polyanion ([−]), expressed as $Z=\frac{\left[+\right]}{\left[-\right]}$ , and varied between 0 to 1.0. It should be noted here that *Z* is not the real charge ratio but a nominal one, as for [−], we simply account for all carboxyl groups, ignoring their actual state of charge. Here, *Z* serves as a comparative and operational parameter rather than an absolute direct measure of electrostatic stoichiometry. The pH values of the pure NaCMC solution (i.e., at *Z* = 0) and the stock PDADMAC solution were ∼7.14 and ∼5.8, respectively. To maintain a constant polyanion concentration of 5 g/L across all samples, the protocol was initiated by introducing the required amount of polyanion into a glass vial, followed by the addition of an appropriate amount of solvent (water) to achieve the desired final polyanion concentration. Subsequently, a prescribed amount of polycation stock solution (PDADMAC), as per the target charge ratio, was meticulously added drop by drop to the solution within the glass vial while sustaining robust stirring to prevent any potential precipitation due to local neutralization of the polyelectrolytes. A photo of the formed IPEC sample in glass vials is shown in Figure SI-1 (in the Supporting Information). This complexation process was accompanied by a gradual pH decrease, from ∼7.14 to ∼6.55 (Figure SI-2 in the Supporting Information), attributed to proton release resulting from interactions between the carboxylate groups (−COO^–^) of NaCMC and the quaternary ammonium groups (−N^+^(CH_3_)_2_-) on PDADMAC. Since the change in the pH of the formed complex is associated with the addition of polycations, it is practically difficult to decouple the actual degree of dissociation of NaCMC as a function of the amount of polycation added to the solution to reach a desired charge ratio value. Thus, the nominal charge ratio *Z* serves better as a comparative and operational parameter for this system. For neutron scattering measurements, all samples were prepared in D_2_O solvent; otherwise, H_2_O was used as solvent. Here, it is noteworthy that approximately 0.08% (in the case of *Z* = 0.1) to 0.8% (in the case of *Z* = 1.0) of H_2_O remained in the final solution (used for neutron scattering characterization) because the supplied PDADMAC solution (20 wt %) was based on H_2_O.

### 2.3 ζ-Potential

ζ-Potential measurements on IPECs were performed using a Malvern ZetaSizer (ZS90), which uses light scattering (He–Ne laser with a wavelength of 633 nm) to measure the electrophoretic mobility of the colloidal particles in dispersion under an applied electric field. The IPEC samples were diluted 10 times and equilibrated at 20 °C for the measurements. The ζ-potential (ζ) values can be calculated from the measured electrophoretic mobility (μ_E_) using the following relation (eq 1)­

1 $$\zeta =\frac{3\eta \mu_{E}}{{2\varepsilon }_{0}\varepsilon_{r}f\left(\kappa a\right)}$$

where “η” is the viscosity, “ε_0_” and “ε_r_” are the permittivity of vacuum and the relative permittivity of the medium, respectively. *f*(κa) is the Debye factor, which was taken as 1.5, applicable for particles much larger than the screening length according to the Smoluchowski approximation. The measurements were conducted in quintuplicate.

### 2.4 Light Scattering

Static (SLS) and dynamic light scattering (DLS) experiments were simultaneously carried out on IPECs using a CGS-3 Compact Goniometer System (ALV GmbH, Langen, Germany), equipped with a He–Ne laser (wavelength λ_L_ = 632.8 nm). For SLS measurements, the scattering angle 2θ_L_ was varied from 25° to 144° to access a wave-vector transfer range (*Q*_L_) of ∼0.006–0.025 nm^–1^. The magnitude of *Q*_L_ was calculated using $Q_{L}=\frac{4\pi n_{s}\;\sin\left(\theta_{L}\right)}{\lambda_{L}}$ , where “*n*_s_” is the refractive index of the solvent (H_2_O). The SLS intensity (*I*_L_) was corrected and normalized using the following equation (eq 2)­

2 $$I_{L}\left(Q_{L}\right)=\frac{{\left(\frac{\mathrm{CR}1}{I_{\mathrm{mon}}}\right)}_{\mathrm{sample}}-{\left(\frac{\mathrm{CR}1}{I_{\mathrm{mon}}}\right)}_{\mathrm{solv}}}{{\left(\frac{\mathrm{CR}1}{I_{\mathrm{mon}}}\right)}_{\mathrm{tol}}}\times R_{\mathrm{tol}}$$

where “CR1” denotes the average recorded scattered intensity normalized to the laser intensity, “*I*_mon_”. Toluene was used with its known Rayleigh ratio (*R*_tol_) to calibrate the instrument.^40^ The scattering intensity distribution over *Q*_L_ was analyzed using the Guinier approximation^41^ (eq 3)­

3 $$I_{L}\left(Q_{L}\right)≅I_{0}\;\exp\left(-\frac{Q_{L}^{2}R_{g}^{2}}{3}\right)$$

The approximation holds valid for *Q*_L_ *R*_g_ ≪ 1 to estimate the radius of gyration (*R*_g_) and the forward scattering intensity, *I*_0_ (*Q*_L_ → 0). The apparent molecular weight (*M*_w_^app^) of the aggregates was obtained from the estimated value of forward scattering using the following equation (eq 4)­

4 $$M_{w}^{\mathrm{app}}=\frac{I_{0}}{\mathrm{cK}}$$

where “*c*” is the solute concentration and “*K*” is the optical constant related to the specific refractive index increment, $\frac{dn}{dc}$ via the following equation (eq 5)­

5 $$K=\frac{{4\pi }^{2}}{N_{A}\lambda_{L}^{4}}{\left(n_{0}\frac{dn}{dc}\right)}^{2}$$

Here, $\frac{dn}{dc}$ of the IPEC solution was determined as a weighted average from the $\frac{dn}{dc}$ of the individual polymer reported elsewhere^42,43^ (for further details, see S3 in the Supporting Information). “*N*_A_” denotes Avogadro’s constant.

DLS measures the fluctuations in the scattered light intensity of aggregates undergoing Brownian motion. The intensity fluctuations over time (*t*) were measured at a scattering angle (2θ_L_) 90° using an ALV-7004/FAST real-time digital correlator to compute the intensity autocorrelation function, *g* ^(2)^(τ), as a function of delay time (τ) as follows (eq 6)­

$$g^{\left(2\right)}\left(\tau \right)=\left\langle I_{L}\left(t\right)\right\rangle \langle I_{L}\left(t+\tau \right)\rangle /{\left\langle I_{L}\left(t\right)\right\rangle }^{2}$$ 6

Here, ⟨*I*_L_(*t*)⟩ denotes the temporal average of the scattering intensity. We used the CONTIN^44^ method to analyze the field autocorrelation function and found a bimodal exponential to be a suitable choice for the analysis. More details are provided in Supporting Information. *g* ^(2)^(τ) was then fitted using a bimodal exponential, as shown in eq 7, to determine the inverse relaxation times (Γ_i_).

7 $$g^{\left(2\right)}\left(\tau \right)-1=\sum_{i=1}^{2}A_{i}\cdot \exp\left(-2\Gamma_{i}\tau \right)$$

Here, “*A_i_*” is a scaling factor. Γ_1_ and Γ_2_ can be directly related to the two modes of relaxation vis-à-vis the diffusion coefficients, *D*_1_ and *D*_2_ (Γ*_i_* = *D_i_* ·*Q*_L_^2^) of the small aggregates, and their hierarchical assemblies. The hydrodynamic radius (*R*_H_) of the particles, assuming spherical aggregates, can be estimated using the Stokes–Einstein relation^45^ (eq 8)­

8 $$R_{H,i}=\frac{k_{B}T}{6\pi \eta D_{i}}$$

where “*k*_B_” is the Boltzmann constant, “*T*” is the temperature, and “η” is the viscosity of the solvent. Here, “*R*_H,1_” represents the hydrodynamic radius (*R*_H_) of the IPECs, and “*R*_H,2_” represents the hydrodynamic radius (*R*_LC_) of the large-sized hierarchical cluster present in the solution. It is noteworthy that all IPEC samples were diluted 10-fold to ensure adequate transmission of light during measurements. Each measurement was repeated three times. The total intensity was recorded for 60 s per acquisition. All measurements were carried out the next day of sample preparation.

### 2.5 UV–Vis Spectroscopy

The transmission of the IPEC samples was measured using a Jasco V-630 ultraviolet (UV–vis) spectrophotometer at a fixed wavelength of 500 nm. The supernatant of each IPEC sample was transferred to a polystyrene cuvette with an optical path length of 10 mm for measurement. Each measurement was carried out in quintuplicate at 20 °C. A cuvette filled with solvent (water) was used as a “blank” to normalize the transmission value. Using Beer–Lambert’s law, the turbidity (τ) of the IPEC solution is calculated from the measured transmission (*T*) value using the following equation (eq 9)­

9 $$\begin{array}{l} T=\frac{I_{M}}{I}=\exp\left(-\tau d\right)\\ \mathrm{or},\tau =\frac{2.3\cdot \log\left(T\right)}{d} \end{array}$$

where “*I*_M_” and “*I*” denote the measured and incident intensities of light, respectively, and “*d*” is the optical path length.

### 2.6 Small-Angle Neutron Scattering

Small-angle neutron scattering (SANS) characterization of the IPECs was carried out at the Institut Laue-Langevin (ILL), Grenoble, France, using a D33 massive dynamic *Q*-range small-angle diffractometer.^46^ The IPEC solutions were gently filled into quartz cuvettes (Hellma, QS type) with a 2 mm optical path length, followed by mounting in a sample changer rack. The sample changer rack was thermostated using a water bath, and all SANS measurements were performed at 20 °C. A wide *Q*-range (0.0015–4.4 nm^–1^) was achieved by recording the scattered neutrons at two neutron wavelengths (λ ∼ 12.0 and 4.65 Å, with a full width at half-maximum (FWHM) of ∼10%). Here, “*Q*” is the modulus of the wave-vector transfer, calculated as $Q=\frac{4\pi \;\sin\left(\theta \right)}{\lambda }$ , where “2θ” and “λ” are the scattering angle and neutron wavelength, respectively. The neutron beam collimation length was 12.8 m, and the rear two-dimensional (2D) detector was placed 13.3 m from the sample. Additionally, four front panel detectors were used at 1.7 m (for the top and bottom panels) and 1.885 m (for the left and right panels) from the sample. The raw scattering data were normalized by the monitor counts and corrected for the flat field, relative detector efficiency (variation between detector panels), and background noise (measured with sintered ^10^B_4_C at the sample position). The empty cell and solvent background contributions were measured using an empty cuvette and a cuvette filled with D_2_O, respectively. The scattering backgrounds were subtracted from the sample scattering signal with measured transmission factors, followed by conversion to the absolute scale intensity. For conversion to the absolute scale, the flux of an attenuated direct beam was measured on the rear detector with a known attenuation coefficient. The scattered intensity was azimuthally averaged to obtain one-dimensional (1D) scattering profiles. All data reduction processes were carried out using the GRASP package.^47^

## 3 Results and Discussion

### 3.1 Phase Behavior of the Formed IPECs

We first examined the phase behavior of the formed IPECs as a function of the mixing charge ratio before conducting their structural characterization. The stock solutions of PDADMAC and NaCMC appeared to be visually transparent. To assess the phase behavior, the *Z* ratio was varied from 0.0 to 2.0 to determine the phase behavior of the samples. The appearance of the formed suspensions at different times after their preparation is shown in Figure SI-1 in the Supporting Information, while the turbidity measurements are presented in Figure 1a. At molar charge ratios up to 0.4, we observed a monophasic colloidal dispersion with a slight increase in the turbidity value. As the charge ratio was varied further from 0.5 to 1.0, the solution became significantly more turbid, reaching a maximum turbidity at *Z* = 0.9. At charge equimolarity (i.e., *Z* = 1.0), phase separation was observed, with a dense white polymer-rich phase observed at the bottom of the vial, while the supernatant still appeared to be turbid. Beyond *Z* = 1.0, the solution underwent immediate phase separation via precipitation, leaving a relatively clear supernatant. It might be noted that we observed that samples become monophasic again for *Z* > 2.5, but this region of high *Z* values was not studied in further detail by us.

**Figure 1:**
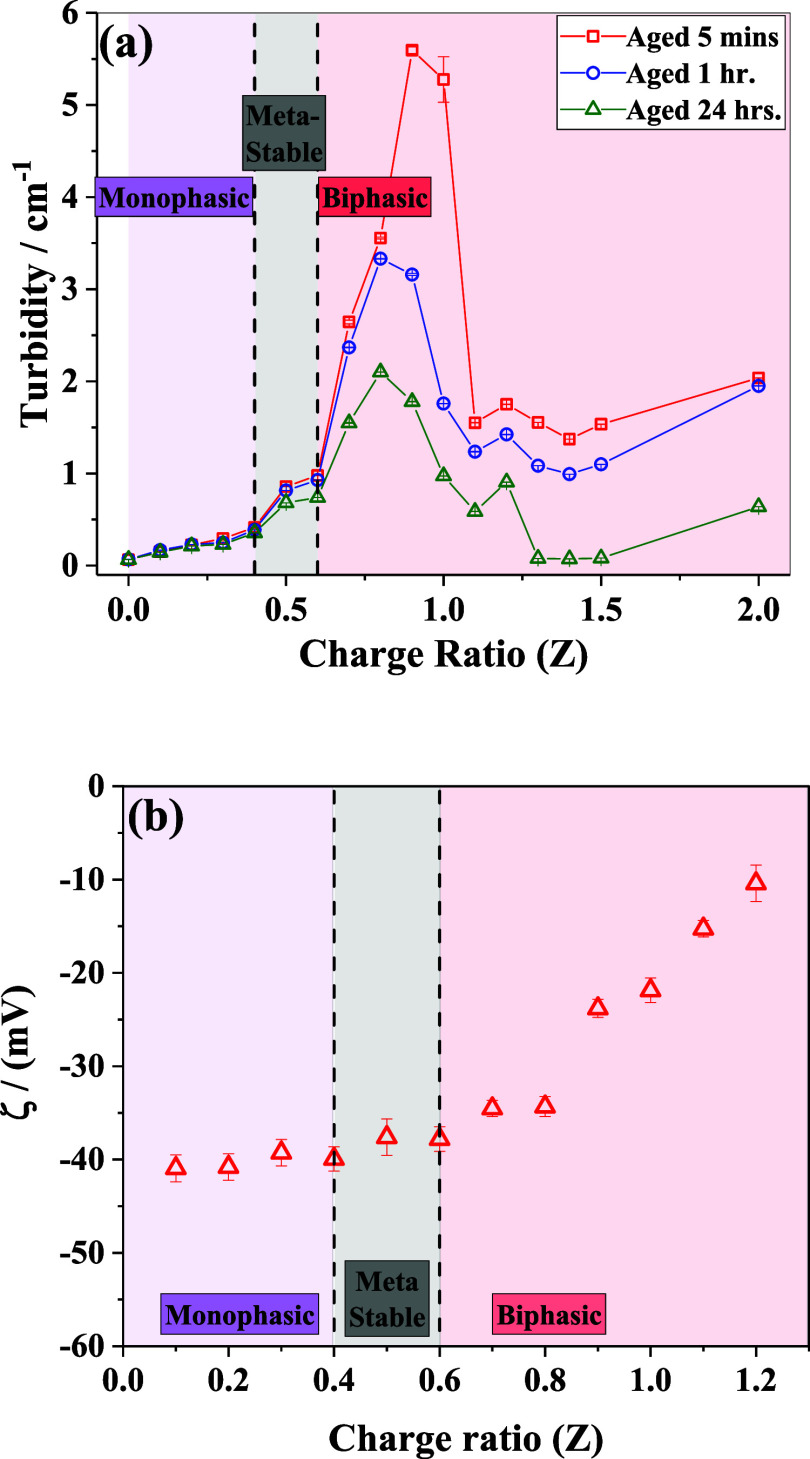
(a) Turbidity of the suspension as a function of the mixing charge ratio at different aging times after preparation. (b) ζ-potential as a function of charge ratio *Z*. The vertical dashed lines represent the macroscopic phase boundaries of the IPECs.

To evaluate the stability of the prepared samples, turbidity measurements were carried out over different aging times, as depicted in Figure 1a. Over 24 h, the samples with charge ratios up to 0.4 remained stable, whereas those with *Z* = 0.5 and 0.6 showed a ≈20–24% decrease in turbidity, likely due to sedimentation in the gravitational field, but it could also be related to the onset of macroscopic phase separation. For samples with *Z* > 0.6, phase separation was evident after 24 h. The ζ-potential measurements (Figure 1b) carried out the next day of sample preparation allowed us to evaluate the colloidal stability in terms of the surface charge of the formed complexes. The complexes exhibited a negative surface charge, as expected for the polyanion-excess region. The ζ-potential remained well below −40 mV, implying a charge-stabilized colloidal suspension for mixing charge ratios up to 0.8. In the vicinity of the charge equimolarity region (*Z* = 0.9 to 1.2), the ζ-potential value still remained negative, suggesting the supernatant contained polyanion-rich complexes (and the precipitate likely contained an excess of polycations). Here, it is noteworthy that PDADMAC possesses a quaternary ammonium group on every repeating unit, effectively contributing one positive charge for approximately every three backbone atoms. In contrast, NaCMC contributes on average one negative charge per ∼six backbone atoms. This inherent difference in the linear charge density suggests that excess PDADMAC is required to achieve complete charge neutralization. This is in agreement with the turbidity measurements (Figure 1a) and the visual appearance of the IPEC solutions (S1 in the Supporting Information). At *Z* > 1.0, while the turbidity of the supernatant decreases significantly, the maximum precipitation occurs at *Z* ≈ 1.3–1.5 due to the formation of large polymer-rich complexes, implying that local charge density plays a crucial role in the complexation behavior.

### 3.2 Structural Analysis of Macromolecular Aggregates by Light Scattering

The SLS profiles are shown in Figure SI-3 in the Supporting Information. The Guinier approximation (eq 3) was fitted to the experimental profiles to extract the mass-averaged structural length scale, i.e., the radius of gyration (*R*_g_) of the macromolecular aggregates. The variation in *R*_g_, as depicted in Figure 2a, shows no significant change up to *Z* = 0.4. At *Z* = 0.5, the aggregate size increased by ∼40%, indicating the formation of larger macromolecular aggregates in solution. This also corroborates the increase in the macroscopic turbidity of the solution, as discussed earlier. However, as the charge ratio approached equimolarity, a slight decrease in the aggregate size was observed, which is primarily associated with the onset of phase separation. At near charge equimolarity, the larger-sized hierarchical aggregates start separating out from the supernatant of the solution, resulting in a decrease in the observed aggregate size. Additionally, by extracting the *I*_0_ value from the Guinier-law fitting, we estimated the apparent molecular weight (*M*_w_^app^) of the formed macromolecular aggregates using eq 4, as shown in Figure 2a. We observed a continuous increase in the molecular weights of the formed IPECs up to *Z* = 0.4 without an appreciable change in their structural length scale. This signifies either the formation of a higher number of complexes with similar size and polymer density, or a significant increase in the polymer density within the formed complexes, resulting in more compact local structures. The first possibility can be ruled out by the interpretation of the local structure of the aggregates, as revealed by the SANS analysis in the subsequent section. However, at *Z* > 0.5, the variation in molecular mass somewhat follows the same trend as the radius of gyration, suggesting no significant change in the polymeric density of the formed complexes. The estimated parameters from the SLS are listed in Table 1.

**Figure 2:**
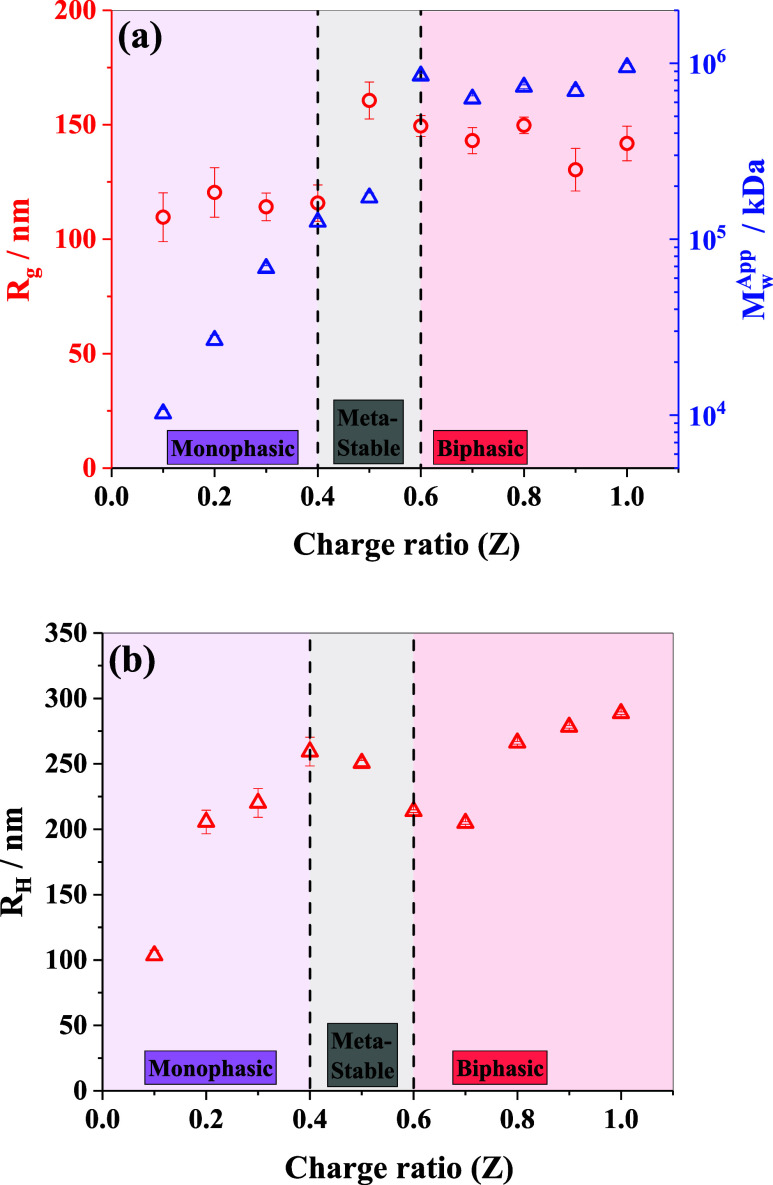
(a) Variation in the estimated radius of gyration (left scale) and apparent molecular weights (right scale) obtained from the SLS data as a function of molar charge ratio. (b) Variation in the hydrodynamic radius of the formed complexes at different molar charge ratios. Measurements were taken 24 h after sample preparation. The vertical dashed lines represent the macroscopic phase boundaries of the IPECs.

**Table 1:** Estimated Parameters from Light Scattering for Samples with Different Charge Ratios *Z* ^a^

sample	parameters obtained from SLS					
*Z*	*R*_g_/nm	*I*_0_/cm^–1^	*M*_w_^App^/kDa	*R*_H_/nm	*R*_LC_/μm	*R*_g_/*R*_H_
0.1	110 ± 11	0.0013 ± 0.0001	1.0 × 10^4^	103 ± 4	0.9	1.1
0.2	120 ± 11	0.0033 ± 0.0002	2.7 × 10^4^	206 ± 9	1.5	0.6
0.3	114 ± 6	0.0085 ± 0.0003	6.8 × 10^4^	220 ± 11	1.8	0.5
0.4	116 ± 8	0.015 ± 0.001	1.3 × 10^5^	259 ± 11	1.5	0.4
0.5	161 ± 8	0.021 ± 0.001	1.7 × 10^5^	251 ± 2	1.6	0.6
0.6	149 ± 5	0.104 ± 0.003	8.5 × 10^5^	214 ± 1	1.6	0.7
0.7	143 ± 6	0.076 ± 0.003	6.3 × 10^5^	204 ± 1	1.8	0.7
0.8	150 ± 4	0.089 ± 0.002	7.3 × 10^5^	266 ± 1	1.9	0.6
0.9	130 ± 9	0.084 ± 0.005	6.9 × 10^5^	278 ± 1	2.0	0.5
1.0	142 ± 8	0.114 ± 0.006	9.5 × 10^5^	289 ± 1	2.1	0.5

a *R*_g_, radius of gyration; *I*_0_, forward scattering; *M*_w_^app^, apparent molecular weight; *R*_H_, hydrodynamic radius; *R*_LC_, large cluster radius; and *R*_g_/*R*_h_, ratio.

In addition to the SLS data analysis, we now focus on the DLS measurements (Figures SI-4 and SI-5), which provide insight into the diffusion behavior of the formed complexes. It is noteworthy that DLS captures time-averaged diffusion behavior and is highly sensitive to the presence of larger aggregates. Even a few large aggregates can skew the data significantly. Moreover, the diffusion characteristics of IPECs are strongly influenced by loosely bound chain segments and the associated solvent layer, which contribute to hydrodynamic drag, and for very asymmetric mixing ratios also by electrostatic interactions. Here, we determined the effective diffusion coefficient, *D*, and the hydrodynamic radius, *R*_H_, using eqs 7 and 8, respectively, by assuming spherical morphology. The variation in *R*_H_ with the charge ratio (*Z*) is shown in Figure 2b, revealing a gradual increase up to *Z* = 0.4. Between *Z* = 0.5 and 0.7, the value of *R*_H_ decreases slightly before increasing again. The ratio of the radius of gyration to the hydrodynamic radius (*R*_g_/*R*_H_), presented in Table 1, remains between 0.4 and 0.7 across the charge ratios, except at *Z* = 0.1. This indicates that the formed IPECs are consistent with structures like dense polymer globules, suggesting that the components are well integrated within the assembled structures rather than forming loosely aggregated or extended conformations (however, it should be noted that the ratio *R*_g_/*R*_H_ is not very instructive for noncompact aggregates, as it will in detail depend also strongly on the effective solvent binding). In addition, we observed the formation of hierarchical clusters in the range of 1–2 μm (see Table 1), as indicated by the slower mode of the diffusion coefficient.

### 3.3 Structural Analysis of Macromolecular Aggregates by SANS

For obtaining further structural insight into the formed IPECs, we now discuss the small-angle neutron scattering (SANS) results. SANS is a formidable nondestructive technique that maps the neutron scattering length density fluctuation of inhomogeneities of mesoscopic length scales into the reciprocal space.^41,48,49^ It is often used to probe the internal structure of polymeric systems under native conditions,^50^ and it overcomes statistical bias unlike electron microscopy. Thus, it is prudent to use SANS as a pivotal characterization tool for a comprehensive understanding of the hierarchical and complex structures of IPECs with nanoscale precision. However, it is noteworthy that the analysis of SANS data for such complex macromolecular aggregates is not trivial. IPECs often exhibit broad size distributions and complex shapes; thus, common geometric models may fail to describe such systems with precision and can lead to poor and unreliable interpretations. To overcome this difficulty, we here discuss the integration of both model-independent and model-dependent approaches and thereby corroborate the analyzed results by combining a set of analysis approaches.

#### 3.3.1 Model-Independent Structural Analysis

Figure 3 depicts the SANS profiles collected on the IPECs with varying charge ratios (0 to 1.0) of cationic (PDADMAC) to anionic (NaCMC) polyelectrolyte charges, keeping the mass of anionic polyelectrolyte CMC at a constant 5 g/L. The SANS profiles in Figure 3 show that immediately upon adding PDADMAC to NaCMC, the scattering pattern changes systematically with an appreciable increase in intensity at lower and intermediate *Q* (∼0.01–0.5 nm^–1^) as well as a slight decrease in the intensity at higher *Q* (∼0.5–3.0 nm^–1^), thereby indicating a structural transformation from individual polyelectrolyte chains to more compacted aggregates.

**Figure 3:**
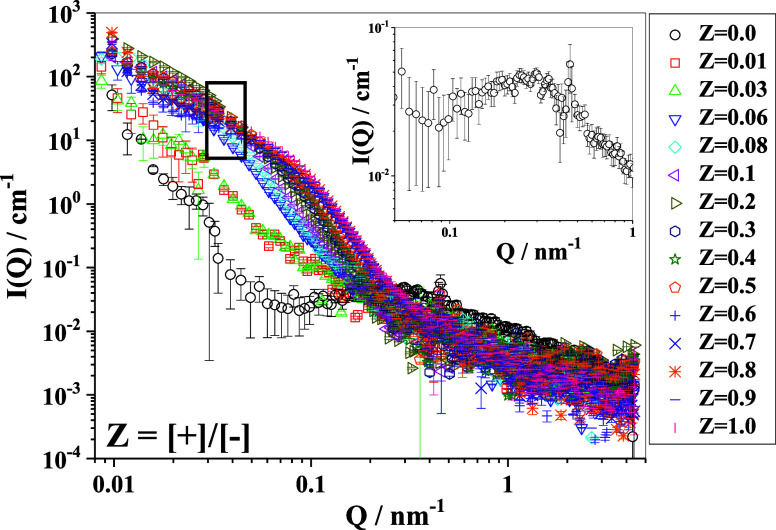
SANS profiles of the formed IPECs at different charge ratios (at 20 °C). The rectangular box indicates the region of the iso-intensity crossover. The inset shows the correlation peak observed for pure NaCMC (*Z* = 0).

For pure NaCMC, one observes the characteristic correlation peak for polyelectrolytes at ∼0.25 nm^–1^ (inset of Figure 3), indicating an average spacing of ∼24 nm between the NaCMC chains, which is in very good agreement with the expected spacing for rather stretched NaCMC chains (for details, see S4 in the Supporting Information). Very interestingly, this correlation peak vanishes in the formed macromolecular complexes upon the addition of very small amounts of polycation, such as *Z* = 0.01. Apparently, as low as 1% oppositely charged polyelectrolyte was sufficient to screen the Coulombic repulsion of the polymeric chains of NaCMC rather completely and lead to substantial structural rearrangement, as seen mostly in the *Q*-range lower than the original correlation peak. Given that the initial NaCMC solution has a pH of 7.14 and the p*K*_a_ of CMC is approximately 4,^51^ CMC must be ionized well above 50%. Consequently, compensating the CMC charge with only 1–2% PDADMAC is insufficient to significantly reduce its overall charge. Therefore, the disappearance of the structure factor peak cannot be attributed to charge neutralization. Instead, this is likely due to the disruption of the polyelectrolyte ordering of CMC induced by the presence of PDADMAC. A plausible explanation is that PDADMAC forms bridges between different CMC chains, thereby substantially altering their degree of ordering and largely reducing their repulsive interaction. If we observe the scattering profiles in Figure 3 more closely, the scattering intensity systematically increases in the range of *Q* = 0.04–0.2 nm^–1^ with increasing *Z*, while at lower range of *Q* = 0.01–0.04 nm^–1^, one observes the highest intensity at *Z* = 0.2, followed by a systematic decrease to the lowest value at *Z* = 0.6, and subsequently, it increases again with further increase of *Z*. It is interesting to note that scattering profiles show an iso-intensity crossover at *Q* ∼ 0.05 nm^–1^, indicating formation of local globular domains that are embedded in a larger network structure and becomes more pronounced with increasing *Z*. This interpretation is shown in a graphical format in Figure SI-6 in the Supporting Information, where we plot the intensity at *Q* = 0.01, 0.05, and 0.10 nm^–1^. Samples at *Z* = 0.9 and 1.0 exhibited slight phase separation during SANS measurements; however, since no significant change in scattering intensity was observed at high *Q*, the total polymer concentration was used for the SANS data analysis. The remaining samples were homogeneous throughout the cuvettes.

In order to better visualize the above-mentioned changes in the scattering data, we represent them in Figure 4a in the form of Kratky plots, where the square of the momentum transfer (*Q* ^2^) multiplied by the intensity of scattering [*I*(*Q*)] is plotted against *Q*, i.e., *Q* ^2^ *× I*(*Q*) vs *Q*. Kratky plots offer valuable insights into the morphological properties of the structures, facilitating a deeper understanding of the conformation of the complexes formed. In particular, Kratky analysis elucidates the extent of molecular folding of a macromolecule.^52^ The bell-shaped curves in Figure 4a indicate the formation of globular compacted structures that become more pronounced with increasing values of *Z*. In the higher *Q* region, the slope of all scattering profiles tends to zero, indicating either the formation of a compact globular structure or the presence of a Gaussian polymeric chain contained within the large aggregates.^8,9^ The position of the peak (Q*) on the Kratky plots provides a tentative estimate of the overall characteristic length scale (*R*_g_) of the structures (Figure 4b), given by the relation Q*·R_g_ ≈ √3 for a typical globular-shaped scattering object.^53^ To evaluate the peak positions, the profiles were fitted to Gaussian curves near the peak position (see S6 in the Supporting Information). As the value of the charge ratio *Z* increases, there is a shift in the peak positions toward higher Q* values, indicating a decrease in the characteristic length scales vis-à-vis the formation of smaller-sized particulate structural units, as depicted in Figure SI-7 in the Supporting Information. Moreover, the increasing peak width, as shown in Figure 4b, suggests an increase in the polydispersity of the size and shape of the formed IPECs. This feature may also be attributed to the presence of larger structural aggregates comprising positional correlations between the formed complexes. At lower charge ratio values, the polycationic polymer complexes may mainly be composed of a diffuse charge-neutralized region surrounded by an electrostatically stabilizing region of excess polyanions. As the charge ratio increases, a lesser amount of uncompensated polyanionic polymers remains on the surface of the complex, resulting in more conformational flexibility owing to a lesser constraint with respect to electrostatic stabilization. This results in the formation of interconnected, smaller units of charge-neutralized local structures.

**Figure 4:**
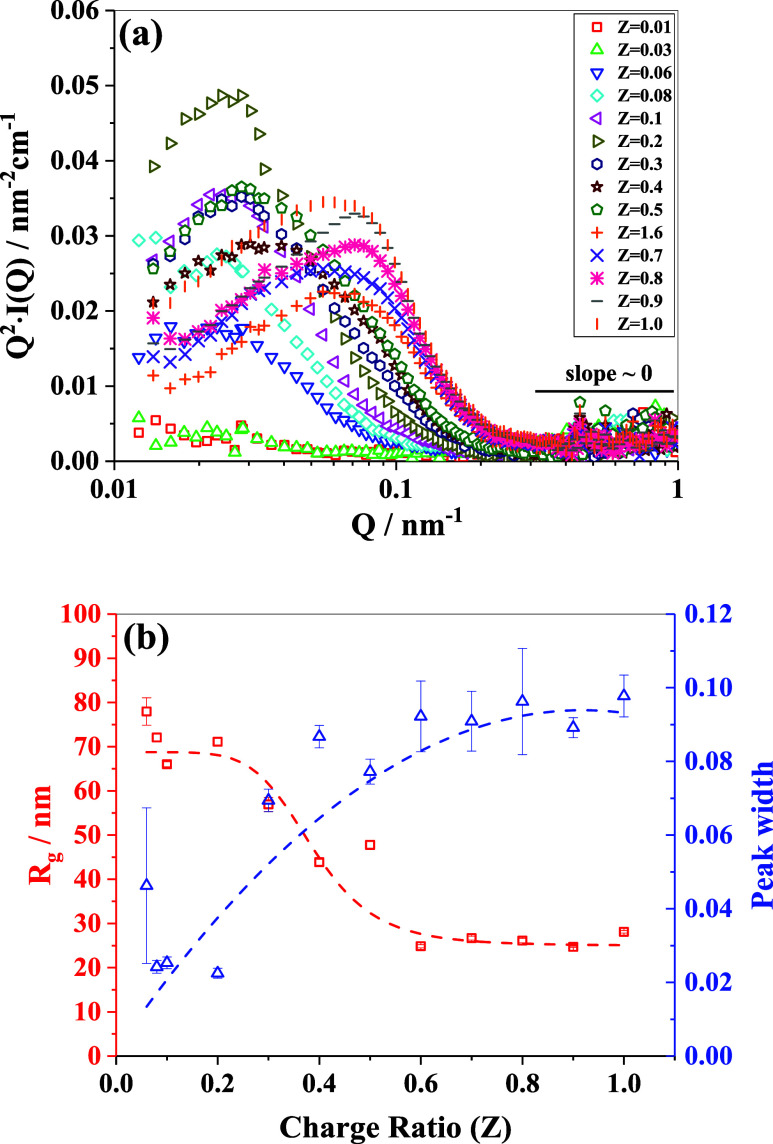
(a) Scattering intensity profiles in the Kratky representation, illustrating the conformational change of the formed macromolecular complexes. (b) Variation in the estimated radius of gyration (left axis) and peak width (right axis) obtained from the Gaussian profile fitting to the Kratky plots. The dotted lines are shown as a guide-to-eye.

For a more detailed structural insight into the size and shape of the formed complexes, pair-distance distribution functions (PDDF) were determined by evaluating the inverse Fourier transform of the experimental scattering profiles using eq 10

10 $$P\left(r\right)=\frac{r^{2}}{2\pi^{2}}\int Q^{2}I_{\mathrm{expt}}\left(Q\right)\frac{\sin\left(\mathrm{Qr}\right)}{\mathrm{Qr}}dQ$$

where *P*(*r*) represents the PDDF and “*r*” denotes the distance between a pair of scattering volume elements (atoms) inside the particle. The PDDF represents the spatial distribution of scatterers within aggregates and contains valuable information about the particle shape. The distance at which *P*(*r*) tends to zero provides an estimation of the maximum aggregate size (*D*_max_). PDDF analysis was carried out using “GNOM”, a built-in software module of the ATSAS software package,^54^ and the results are shown in Figure 5 (the fits in Figure SI-8). The peak of each curve denotes the most probable distance between pairs of scatterers within the aggregate, and it shifts toward lower values of *r* as *Z* increases, indicating a reduction in the aggregate size of the macromolecular complexes. For *Z* values up to 0.5, the relatively slow decrease in the pair-distance probability from its maximum value suggests the formation of loosely connected macromolecular aggregates. However, for *Z* = 0.6 and 0.7, the probability distribution shows a sharp fall from its maximum value as compared to the former cases, thus indicating a morphological transition from a loosely bound structure to a more compact globular shape. Further, as the charge ratio approaches equimolarity, i.e., for *Z* = 0.8, 0.9, and 1.0, the probability distribution shows modulation at higher separation distances with a slower decay rate, suggesting the formation of an extended shape with interconnected multiple domains within the macromolecular aggregate.

**Figure 5:**
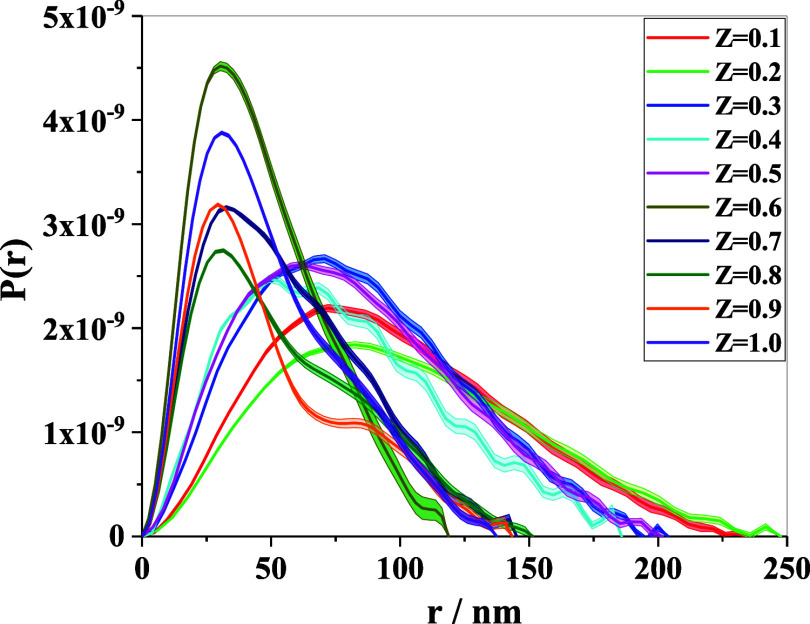
Variation in the estimated PDDF at different charge ratios. All profiles are normalized with respect to the volume of the cluster.

#### 3.3.2 Three-Dimensional Shape Reconstruction of Self-Assembled Aggregates

In continuation of the PDDF analysis, we perform an ab initio reconstruction of the three-dimensional (3D) shape of the formed polymeric complexes using a Monte Carlo-based annealing approach applied to an ensemble of dummy atoms in a confined geometry. The annealing simulation algorithm was implemented using the ATSAS suite^54^ that provides several advanced computational tools, including DAMMIN,^55^ DAMMIF,^56^ and DAMAVER.^57^ The computational model allows the reconstruction of a low-resolution 3D shape of the self-assembled aggregate by keeping the experimental scattering profile as a constraint. Taking the indirect Fourier transform analysis of the experimental scattering profile into account, DAMMIN (Dummy Atom Model Minimization) first creates a search volume equivalent to a sphere of radius *R* = *D*_max_/2, followed by filling it with closely packed dummy atoms of radius *r* ≪ *R*. These dummy atoms or beads serve as individual scattering volume elements belonging to either the particle (i.e., formed polymeric complex) or the surrounding solvent. The partial scattering amplitude is calculated from an arbitrary initial configuration of dummy atoms to obtain the simulated scattering intensity, and then it is compared against the experimental scattering intensity by determining the discrepancy (χ^2^) as follows (eq 11)­

11 $$\chi^{2}=\frac{1}{M-1}\sum_{i}^{M}{\left[\frac{I_{\mathrm{expt}}\left(Q_{i}\right)-I_{s}\left(Q_{i}\right)}{\sigma \left(Q_{i}\right)}\right]}^{2}$$

where “*M*” is the number of experimental data points, *I*_expt_ and *I*_s_ are the experimental and simulated scattering intensity, respectively, and “σ” is the associated error in the experimental intensity measurements. The iterative algorithm modifies the model configuration stochastically to minimize the χ^2^ value and ultimately converges to a low-resolution 3D shape of the macromolecules in solution.

For each scattering profile, 20 sets of independent DAMMIF models are generated for statistical robustness, followed by a variability analysis using the DAMAVER package. DAMMIF is a faster version of DAMMIN that employs a rapid convergence strategy for model fitting, while the DAMAVER tool aligns, filters, and averages the models to produce a consensus shape, removing low-occupancy regions and improving reliability. Finally, DAMMIN refines the low-resolution molecular shape obtained from DAMAVER against the experimental scattering data using a simulated annealing algorithm to find the configuration that best fits the experimental scattering curve while maintaining a compact and connected shape. The good agreement between the experimental and simulated scattering profiles (Figure SI-9 in the Supporting Information) strongly supports the validity of the reconstructed 3D shapes of the IPECs. The constructed dummy atom models for all scattering profiles are visualized in PyMOL,^58^ with front-view projections depicted in Figure 6. For further visualization, three orthogonal projections of the cluster formed at different charge ratios are shown in Figure SI-10 in the Supporting Information. The results indicate that at a relatively lower charge ratio (*Z* < 0.5), where the anionic polymer is in excess of the cationic polymer, self-assembly leads to the formation of a globular shape with lower spherical asymmetry. However, with a further increase in the charge ratio, the polymeric complex exhibits a lumpy morphology comprising multiple aspherical domains. The variation in the maximum cluster size of the formed IPECs is shown in Figure 7, indicating a significant reduction in the size of the macromolecular complex. This morphological transformation indicates that local restructuring takes place in the formed complex owing to the availability of more positive charges for compensation. To quantify the spherical asymmetry of the reconstructed shapes, the aspect ratio (AR) was evaluated using the following formula (eq 12)­

12 $$\mathrm{AR}=\frac{D_{\max}}{2R_{g}}$$

where *D*_max_ is the maximum cluster size and *R*_g_ is the radius of gyration. The variation in the AR values is shown in Figure 8. The estimated AR value remains within 1.53 to 1.57 until a molar charge ratio of 0.5 and subsequently monotonically increases to ∼1.72 with an increase in the molar charge ratio. Here, it is noteworthy that the derived models are illustrative and effective representations, but do not indicate the presence of unique or uniform structural forms across the entire sample. In contrast, they are just an average representative of the real distribution of aggregates of different sizes and shapes.

**Figure 6:**
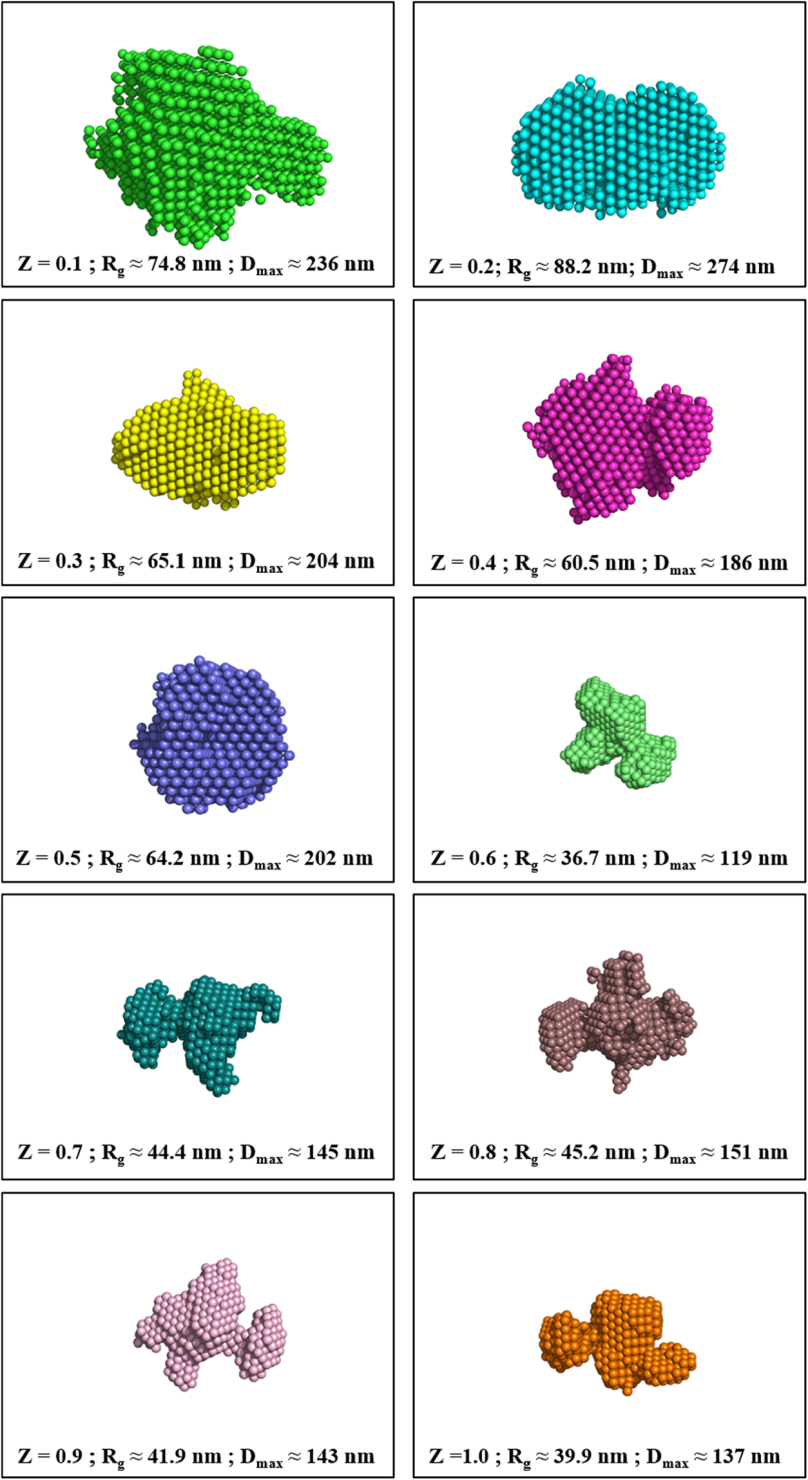
A constructed 3D dummy atom model of the formed aggregates by fitting the experimental scattering profiles. The images are visualized using PyMOL (not to scale).

**Figure 7:**
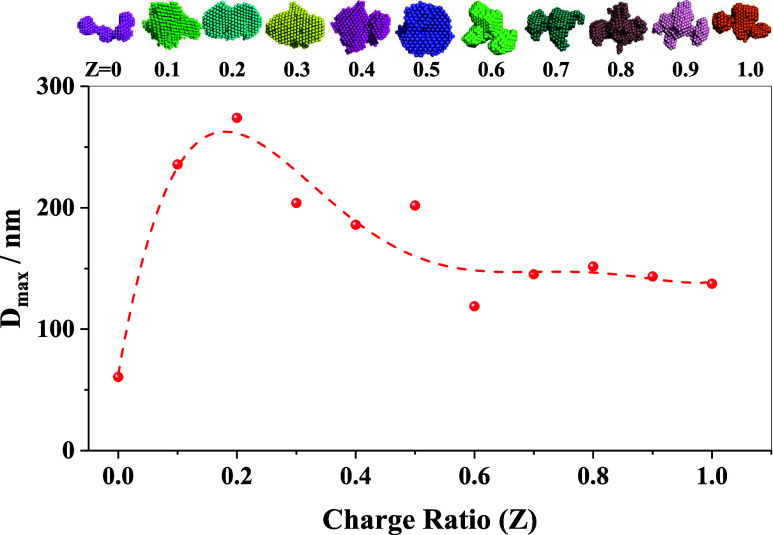
Variation in the maximum cluster size (*D*_max_) with increasing charge ratio. The dotted line represents a “guide-to-eye”. On top, the shapes of the formed aggregates are shown at different *Z* values (not to scale).

**Figure 8:**
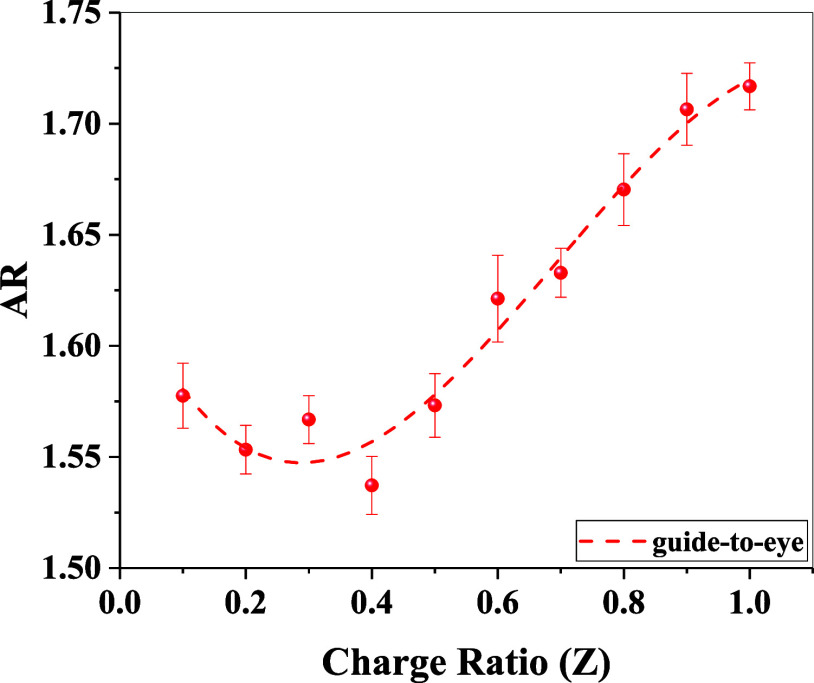
Variation in the aspect ratio with an increase in the mixing molar charge ratio. The dashed line represents a “guide-to-eye” obtained by fitting a 3rd degree polynomial to the data points.

#### 3.3.3 Model-Dependent Quantitative Structural Analysis

After conducting an analysis of the formed macromolecular aggregates using Kratky plots, PDDF, and 3D shape reconstruction, we further conducted a detailed quantitative assessment of the internal structure of the complexes using a mathematically feasible SANS model. For that purpose, the overall scattering intensity can be written as a linear combination of scattering contributions from the formed complexes (*I*_C_), larger structures (*I*_L_), and the free polymer chains (*I*_P_) by neglecting the interaction between these inhomogeneities, provided the spatial scale of density fluctuations of each inhomogeneity is well separated^59^ (eq 13)­

13 $$I\left(Q\right)=I_{C}\left(Q\right)+I_{P}\left(Q\right)+I_{L}\left(Q\right)+B$$

where “*B*” accounts for the incoherent neutron background. It is noteworthy that the higher *Q* (>0.5 nm^–1^) scattering signal can be attributed to the interfacial structure of the formed complexes, a fraction of free polymeric chains that remain in equilibrium with the formed complexes, or, most likely, simply to the internal structure of the complexes. A fraction of unbound polymer is always likely to be present in the system, but it is impossible to decouple these two scattering contributions, especially as the scattering from polymers within the complexes will basically look identical. For simplicity of the SANS model, we attributed the high-Q scattering signal to individual Gaussian chains, expressed as follows (eq 14)­

14 $$I_{P}\left(Q\right)=I_{0,P}2\frac{\exp\left(-u\right)+u+1}{u^{2}}$$

Here, “*u*” is defined as *u* = *Q* ^2^·*R*_g,P_^2^, “*R*_g,P_” is the radius of gyration for the Gaussian polymer chain, and “*I*_0,P_” denotes the forward scattering of the polymeric chains at *Q* = 0. In the fitting model, the value of “*R*_g,P_” was kept constant at ≈1.4 nm for all scattering profiles. At lower *Q* (<0.02 nm^–1^), an upturn in the scattering intensity is observed, particularly for complexes formed with higher charge ratios (*Z* > 0.4), which is attributed to the scattering signals originating from the larger-sized aspects of the hierarchical structure of the aggregates. Here, it is noteworthy that the SLS data revealed an increase in the overall aggregate size of IPECs at *Z* > 0.4, in line with the observed macroscopic turbidity. Since the information about the large-scale structure was outside the SANS experimental *Q*-resolution, a detailed structural analysis of these larger structural features is not possible. The large-scale scattering contribution was considered to be power-law scattering as follows, which is a reasonable choice for fractal aggregates comprised of smaller subunits (eq 15)­

15 $$I_{L}\left(Q\right)=C\cdot Q^{-\alpha }$$

Here, “α” denotes the power-law exponent related to the fractal dimension.^60^ For a 3D mass fractal, α = *D*_m_, mass fractal dimension and 1 < α < 3. For a surface fractal, i.e., an object with surface roughness, α = *D*_s_, surface fractal dimension, and 3 < α < 4. Apart from these two scattering contributions, we now focus on the scattering intensity at the intermediate *Q*-scale beyond the intensity upturn (*Q* > 0.02 nm^–1^). The intensity in the *Q*-range 0.02–0.3 nm^–1^ is primarily dominated by the structure of the aggregated macromolecules within the IPECs. The structure of these macromolecular aggregates can be explained by self-affine random media having a pair correlation^61,62^ (eq 16)­

16 $$g\left(r\right)=\exp\left(\frac{r}{\xi }\right)$$

where *g*(*r*) denotes the pair correlation function with an average correlation length “ξ”. This pair correlation results in a *Q*-dependent intensity distribution as follows (eq 17)­

17 $$I_{C}\left(Q\right)=\frac{I_{0}}{{\left[1+{\left(Q\cdot \xi \right)}^{2}\right]}^{2}}$$

Here, *I*_0_ denotes the forward scattering extrapolated to *Q* = 0. This model assumes smooth interfaces between the inhomogeneities and hence exhibits Porod behavior (*I* ∝ *Q* ^–4^) at large *Q* (*Q*ξ ≫ 1).^61,62^ However, electrostatic domains within IPECs may exhibit diffuse rather than sharp interfaces. To account for this, a generalized Debye–Anderson–Brumberger (gDAB) model is introduced^62^ (eq 18)­

18 $$I_{C}\left(Q\right)=\frac{I_{0}}{{\left[1+{\left(Q\cdot \xi \right)}^{2}\right]}^{\left(3/2+H\right)}}$$

where “*H*” is the Hurst exponent. When *H* → 0.5, the generalized model is reduced to the previous equation. The forward scattering intensity can be written as follows^62,63^ (eq 19)­

19 $$I_{0}={\left(\Delta \eta \right)}^{2}\phi \left(1-\phi \right){4\pi \left(1+2H\right)\xi }^{3}$$

Here, “Δη” denotes the neutron scattering length density difference between the formed complex and solvent (D_2_O), and “ϕ” is the volume fraction of polymer and polymer-bound solvent (for detailed calculations, see S9 in the Supporting Information). The generalized pair correlation can be written as follows^64^ (eq 20)­

20 $$g\left(r\right)=\frac{2}{\Gamma \left(H\right)}{\left(\frac{r}{2\xi }\right)}^{2}K_{H}\left(\frac{r}{\xi }\right)$$

where “*K*_H_” is the modified Bessel function of the second kind and “Γ” is the γ function. The calculated scattering intensity according to the SANS model of eq 13 was fitted to the experimental scattering data based on the “nonlinear least chi-square” fitting using the SASfit program.^65^ An example of the fit for *z* = 0.7, showing all individual contributions, is depicted in Figure 9a, while the fits for all of the different samples are shown in Figure 9b. The fitted parameters are listed in Table 2.

**Figure 9:**
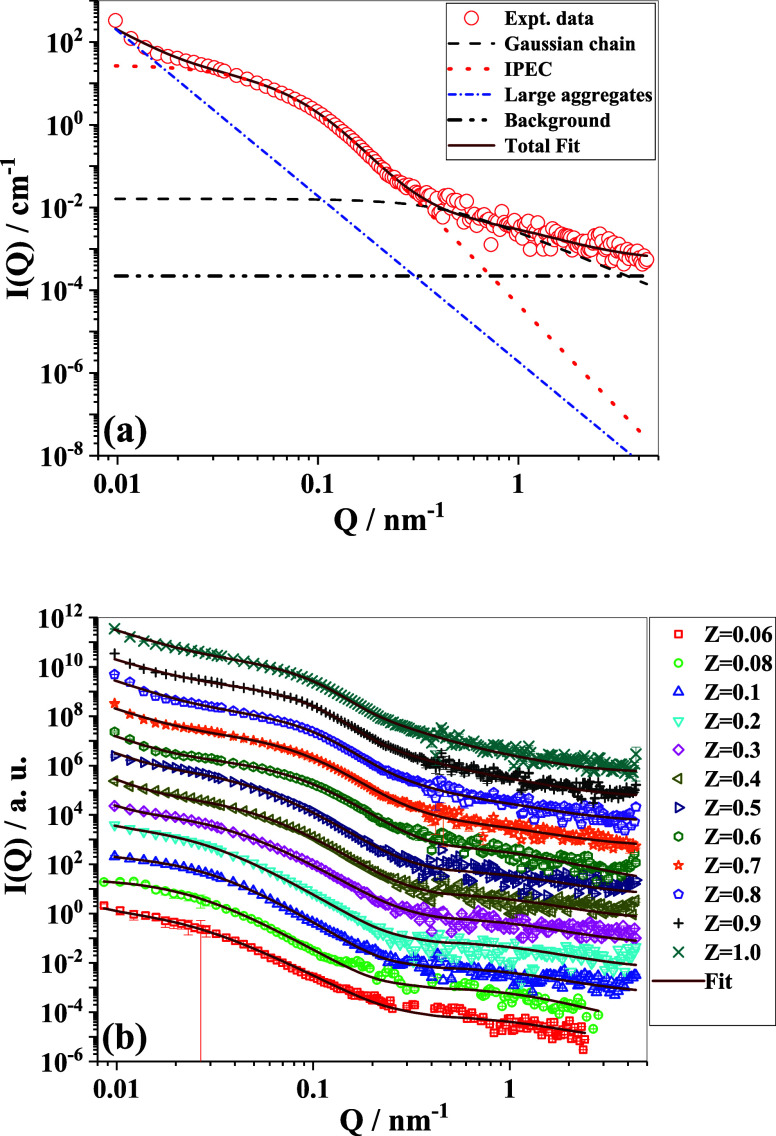
(a) Different scattering contributions used in the three-component SANS model are shown separately to emphasize their relative weightage at different *Q* scales. (b) Experimental SANS data and fits (solid lines) for the complexes with 5 g/L NaCMC and different amounts of added PDADMAC, described by the parameter *Z*.

**Table 2:** Estimated Parameters Obtained from Fitting the SANS Model to the Experimental Data of the Complexes Composed of 5 g/L NaCMC with Varying Amounts of Added PDADMAC, Specified by the Parameter *Z* ^a^

sample	parameters obtained from the absolute intensity scale				estimated positional correlation parameters		large-scale structures	
*Z*	SLD_calc_/cm^–2^	*I*_0,est_/cm^–1^	SLD_hyd_/cm^–2^	Φ_w_ (%)	ξ/nm	*H*	α	*D*_s_ or *D*_m_
0.06	1.68 × 10^10^	133.0 ± 9.9	(6.09 ± 0.23) × 10^10^	94.7 ± 2.5	44.6 ± 2.5	0.50 ± 0.01		
0.08	1.67 × 10^10^	267.6 ± 37.4	(6.01 ± 0.42) × 10^10^	92.9 ± 3.7	43.1 ± 2.4	0.74 ± 0.21		
0.1	1.65 × 10^10^	219.5 ± 10.3	(5.94 ± 0.14) × 10^10^	91.7 ± 2.1	34.2 ± 0.3	0.95 ± 0.11		
0.2	1.59 × 10^10^	332.9 ± 26.7	(5.91 ± 0.24) × 10^10^	91.1 ± 3.7	37.1 ± 0.1	0.87 ± 0.25		
0.3	1.52 × 10^10^	118.1 ± 6.1	(5.94 ± 0.15) × 10^10^	91.8 ± 2.4	27.5 ± 0.3	0.80 ± 011		
0.4	1.47 × 10^10^	54.2 ± 2.6	(5.92 ± 0.14) × 10^10^	91.5 ± 2.2	19.1 ± 0.1	1.05 ± 0.15	3.58 ± 0.08	2.42 ± 0.08 (*D*_s_)
0.5	1.42 × 10^10^	65.7 ± 5.7	(5.91 ± 0.26) × 10^10^	91.3 ± 4.0	19.4 ± 0.1	1.11 ± 0.32	3.41 ± 0.04	2.59 ± 0.04 (*D*_s_)
0.6	1.37 × 10^10^	16.5 ± 0.5	(5.89 ± 0.22) × 10^10^	91.0 ± 3.4	10.7 ± 0.4	1.58 ± 0.11	3.02 ± 0.09	2.98 ± 0.09 (*D*_s_)
0.7	1.33 × 10^10^	17.8 ± 0.3	(5.87 ± 0.08) × 10^10^	90.7 ± 1.3	10.0 ± 0.1	1.93 ± 0.15	2.81 ± 0.01	2.81 ± 0.01 (*D*_m_)
0.8	1.30 × 10^10^	15.2 ± 0.7	(5.81 ± 0.06) × 10^10^	89.6 ± 0.9	7.4 ± 0.1	3.31 ± 0.17	2.78 ± 0.01	2.78 ± 0.01 (*D*_m_)
0.9	1.26 × 10^10^	16.8 ± 0.7	(5.74 ± 0.12) × 10^10^	88.3 ± 1.9	6.2 ± 0.1	4.89 ± 0.18	2.60 ± 0.02	2.60 ± 0.02 (*D*_m_)
1.0	1.23 × 10^10^	18.9 ± 0.8	(5.76 ± 0.12) × 10^10^	88.8 ± 1.8	6.6 ± 0.1	4.70 ± 0.50	2.67 ± 0.01	2.67 ± 0.01 (*D*_m_)

a The parameters derived from the absolute scale include the following: calculated SLD of anhydrous IPECs (SLD_calc_), estimated forward scattering intensity (*I*_0,est_), estimated SLD of hydrated IPECs (SLD_hyd_), and water content in the hydrated IPECs (Φ_w_). Also provided are the correlation length (ξ) and Hurst exponent (H), as well as th*e power-law ex*ponent (α) and fraction dimension of the surface fractal (*D*_s_) or mass fractal (*D*_m_).

The correlation length signifies the extent of the long-range interactions present among the structural units. The estimated correlation length was found to be in the range of ∼19–37 nm for complexes formed at low charge ratios (*Z* < 0.5). While it reached a maximum for *Z* = 0.2, it decreased systematically as *Z* was increased further. The Hurst exponent, which is an estimate of the interfacial interaction between the electrostatic blobs remains almost constant for *Z* < 0.5. This suggests that the polymer chains restructure themselves into smaller units to counterbalance the increasing electrostatic attraction without changing their interfaces. The macromolecular aggregates become more compact with a reduced spatial correlation. As the charge ratio was further increased (*Z* > 0.5), a significant reduction (∼50%) in the correlation length was observed associated with a drastic change in the Hurst exponent value (Figure 10a). The result suggests that as charge equimolarity is approached, the electrostatic blobs overlap with each other, creating a diffuse interfacial interaction. This trend suggests the onset of phase separation and the formation of denser polymer-rich domains.

**Figure 10:**
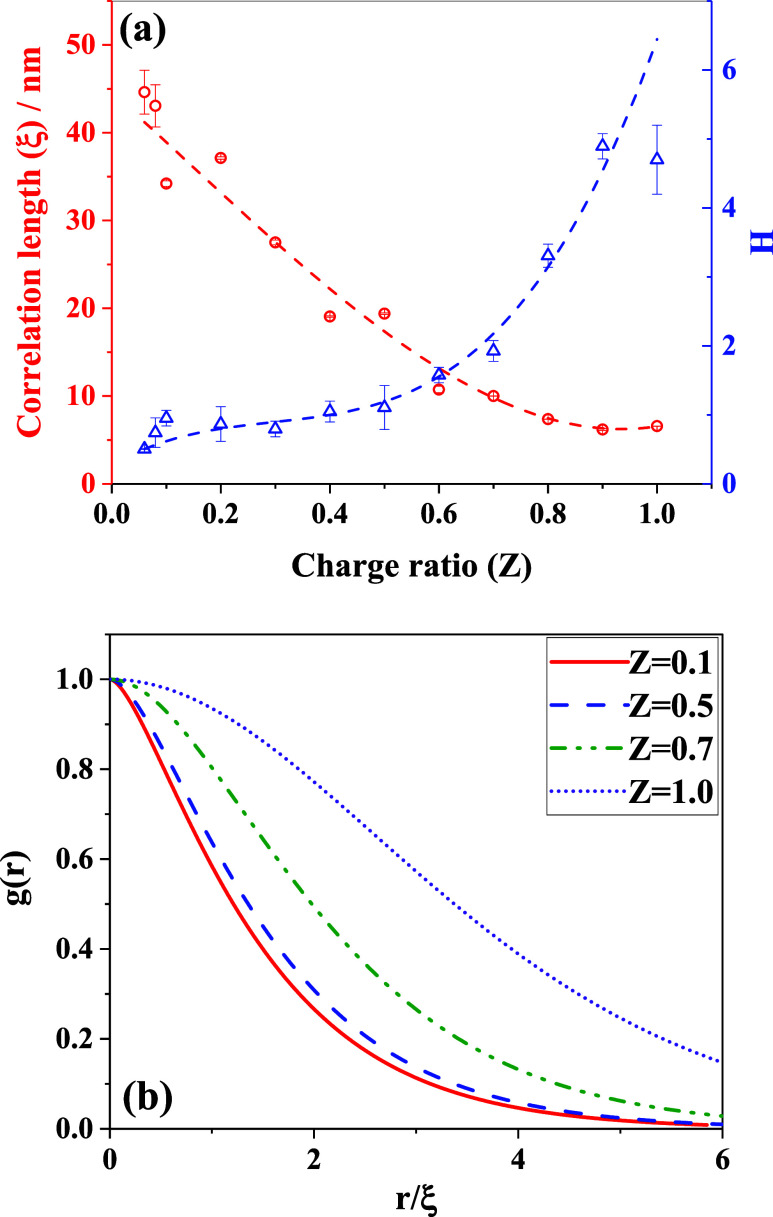
(a) Variation in estimated correlation length between (left scale) the blobs as a function of charge ratio and the variation in Hurst exponent (right scale), signifying the change in interfacial interactions among the blobs as *Z* increases. Dashed lines are shown as “guide-to-eye”. (b) Variation in the calculated pair correlation among the blobs.

Moreover, we inserted the estimated values of the correlation length and Hurst exponent into eq 20 to calculate the pair correlation between the electrostatic blobs, as shown in Figure 10b as a function of the normalized separation distance (*r*/ξ). At lower charge ratios (*Z* < 0.5), the decay of the pair correlation is faster, indicating a short-range structural organization within the aggregate. As the charge ratio was increased, we observed a slower decay in the pair correlation at larger separation distances, implying the formation of a complex structure with inherent long-range interactions. This suggests that at a higher charge ratio, the internal structure of the formed macromolecular aggregate comprises significant interchain entanglements and interactions and has a more extended chain conformation. This could also indicate the formation of a network-like structural aggregate, where the correlation extends to a larger number of structural subunits.

By fitting the SANS model to the absolute intensity scale of the scattering profiles, the probable water content was estimated (details are provided in Supporting Information), and its dependence on *Z* is shown in Figure 11. The macromolecular complexes hold a significant amount of water (∼88–92%) in comparison to the polymer content. Further, it is observed that at higher charge ratios, the structural transformation is associated with a slight release of water molecules (∼3–4%) contained inside the complexes, which means that the polymer concentration increases by ∼50%, i.e., the aggregates become significantly more compacted. Moreover, the value of the estimated power-law exponent decreases from ∼3.6 to ∼2.7 (Table 2), suggesting a gradual transformation of the large-scale structure from a surface fractal network to a mass fractal network as the charge ratio increases. This also suggests the fact that at higher charge ratios with less long-range correlation, smaller subunits tend to aggregate to form larger clusters at the onset of phase separation. A schematic of the aggregation process and structural evolution as a function of *Z* is presented in Figure 12 to visualize the transformation of the internal positional correlation of polymers and the morphological transformation of the overall aggregate.

**Figure 11:**
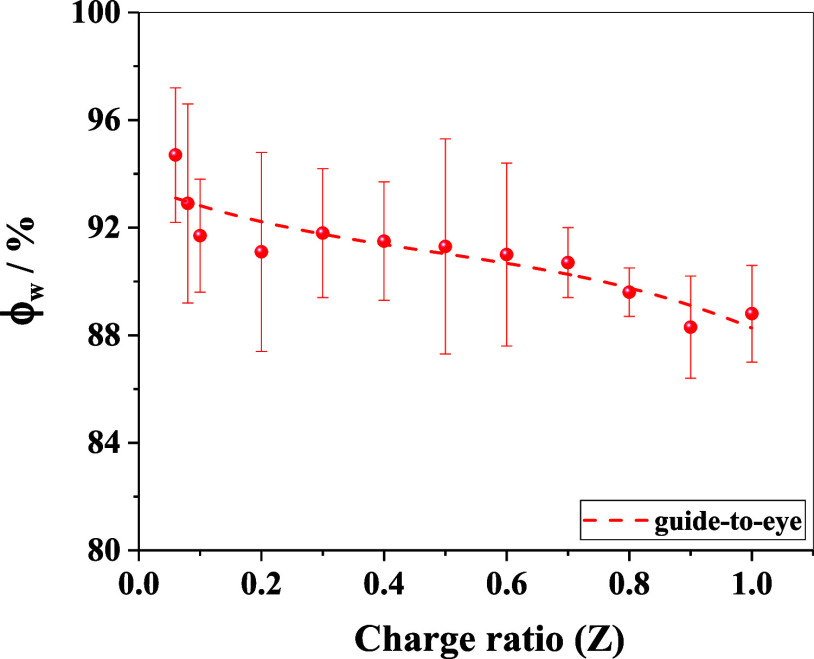
Variation in the hydration of the formed complexes. The restructuring of macromolecular aggregates at a higher charge ratio is associated with the release of hydration.

**Figure 12:**
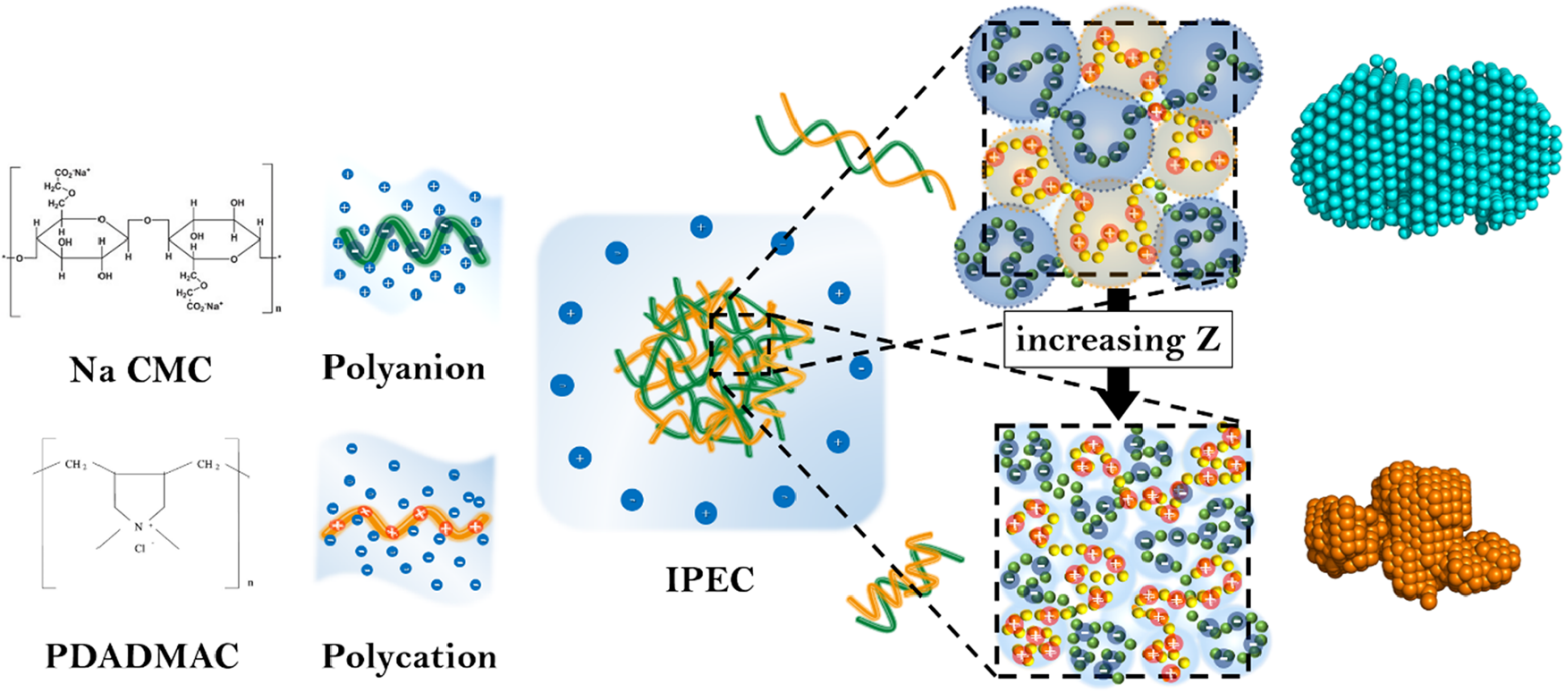
Schematic representation of the transformation of the internal positional correlation and shape of the formed complexes at far and near charge equimolarity.

## 4 Conclusions

In this study, we used small-angle neutron scattering (SANS) and complementary light scattering to characterize the structural properties of interpolyelectrolyte complexes (IPECs) made of oppositely charged polyelectrolytes, sodium carboxymethyl cellulose (NaCMC) and poly­(diallyldimethylammonium chloride) (PDADMAC), in dilute aqueous solution as a function of the mixing ratio. The study reveals how the incremental addition of PDADMAC influences the progression of IPEC formation in a consistent anionic framework of NaCMC at a fixed concentration of 5 g/L. The SANS data were analyzed using a combination of different model-independent and modeling approaches, thereby yielding an independently validated comprehensive structural picture. The maximum aggregate size of ∼270 nm was observed at a charge mixing ratio *Z* of 0.2. The IPECs first became somewhat smaller upon further complexation and then exhibited a significant reduction in size for charge ratios *Z* > 0.5, while at the same time becoming more anisometric. This is a very interesting finding, as one may expect a spherical symmetry for such complexes, which apparently is not the case here. A reason for this behavior may be the rather large difference of the persistence length of polycation and polyanion. However, the overall mass of the IPECs remains rather constant, indicating that the aggregates become more compact, thereby expelling some of the water contained in the rather highly hydrated aggregates (water content ∼90%). This change in the mesoscopic structure is also related to the phase separation observed to take place after a longer time. At *Z* > 0.4, a tendency to form hierarchical aggregates was observed with correspondingly larger total size, which transformed from surface fractal to mass fractal, as revealed by the estimated Porod exponent. The application of the generalized Debye–Anderson–Brumberger (gDAB) model further refines our understanding of the positional correlation of the electrostatic charged domains that undergo diffuse interfacial interactions within the complex.

In summary, it can be stated that the IPECs formed in mixtures of anionic NaCMC and cationic PDADMAC show complex structural evolution as a function of the mixing ratio. The observed structural transitions are influenced not only by *Z* but also by concurrent changes in the polymer concentration and ionic strength. However, a detailed analysis of their SANS data, by combining different analysis approaches, allowed us to discern the details of structural evolution and gain a thorough insight into the structures present, validated by the agreement of the different analysis approaches. Such structural information will be helpful in designing IPECs for further applications in the fields of cosmetics or pharmaceutical formulations, where precise information regarding the structure of IPECs is needed.

## Supplementary Material



## Notes

Visual appearance of IPECs; pH variation of IPEC solutions; light scattering; average spacing of polyelectrolyte chains; absolute SANS intensity variation; Kratky analysis; inverse Fourier transform analysis of SANS data; ab initio modeling of SANS data; and calculation of solvation water in IPECs (PDF)
