# Supplementary material for: Positional Correlation Length-Induced Morphological Transformation of Interpolyelectrolyte Complexes (IPECs) Made of Polysaccharides: The Role of Molar Charge Ratio
Source: Macromolecules. 2025 Sep 26;58(19):10763–77. doi: 10.1021/acs.macromol.5c01621 (PMC12530051; doi:10.1021/acs.macromol.5c01621)
Supplement: Supplementary file 1 [file ma5c01621_si_001.pdf]

Supporting Information

Positional Correlation Length-Induced Morphological Transformation of Interpolyelectrolyte Complexes (IPECs) Made of Polysaccharides: The Role of Molar Charge Ratio

Avik Das[1,2], Sylvain Prévost[2], and Michael Gradzielski[1*]*

[1]Stranski-Laboratorium für Physikalische und Theoretische Chemie, Institut für Chemie, Technische Universität Berlin, D-10623 Berlin, Germany

[2]Large Scale Structures, Institut Laue-Langevin – The European Neutron Source, 71, avenue des Martyrs, 38042, Grenoble, France

[*]Email: avik.das@tu-berlin.de; michael.gradzielski@tu-berlin.de

S1: Visual appearance of interpolyelectrolyte complexes (IPECs) in solution:

The IPECs were prepared in glass vials. A smartphone camera was used to take the photographs of the vials. The visual appearance of the prepared IPEC samples can be seen from the photographs taken after 5 minutes, 1 hour, and 24 hours of sample preparation.

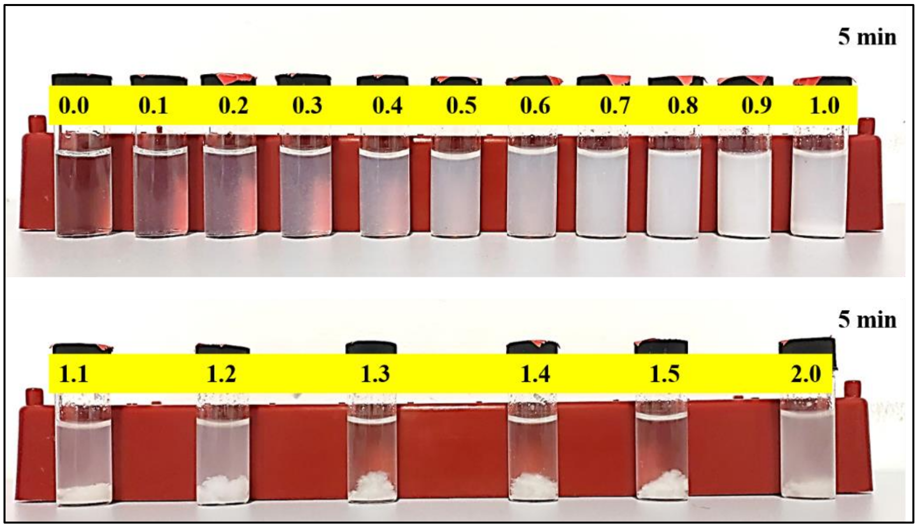

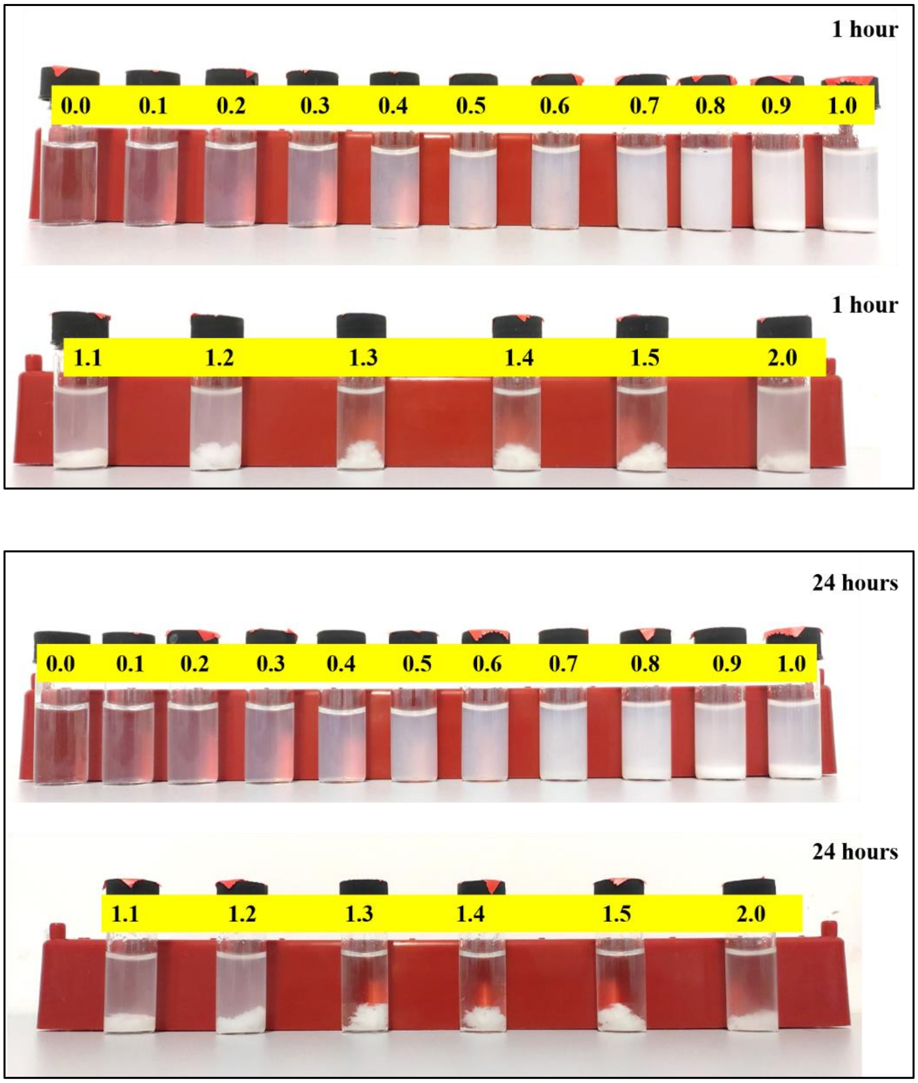

Fig. SI-1*: Visual appearances of the formed IPEC suspensions in glass vials at different aging time.*

S2. Determination of pH of inter-polyelectrolyte complex (IPEC) solutions:

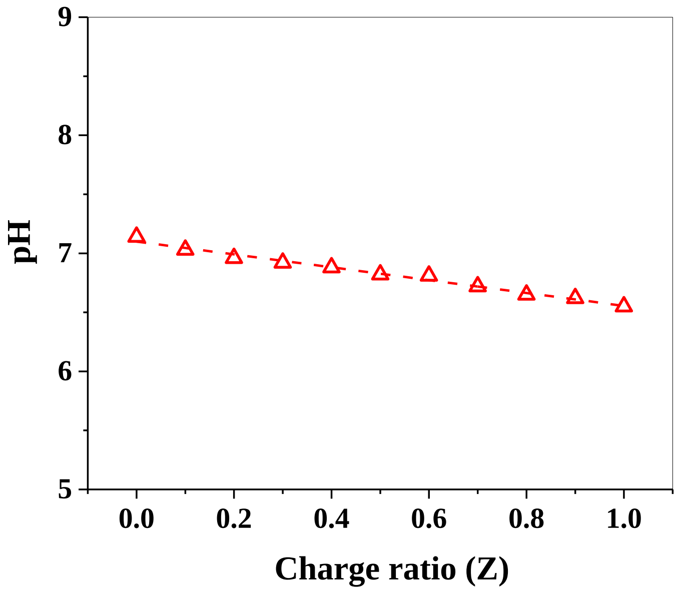

Fig. SI-2*: Variation in pH of IPEC solutions at different charge ratios.*

S3. Light Scattering:

Static Light Scattering (SLS) measurements:

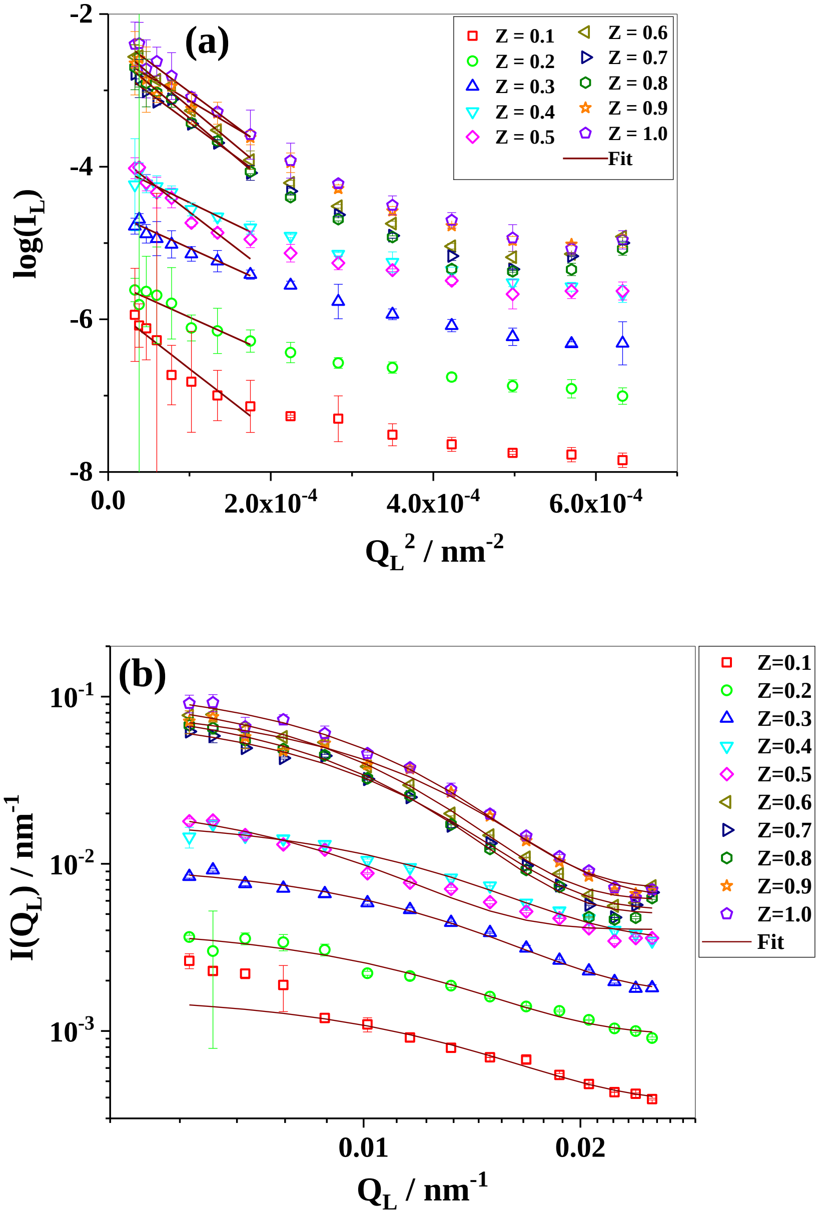

Fig. SI-3: (a) *The Guinier-law fitting to the SLS profiles. (b) The SLS profiles of diluted IPEC solutions in double-logarithmic scale. The solid line represents the fitted curve considering the Guinier approximation.*

**Charge Ratio (Z)**	$\boldsymbol{\Phi_{NaCMC}, \left(\frac{dn}{dc}\right)_{NaCMC}}$ **/ ml. g^{-1}**	$\boldsymbol{\Phi_{PDADMAC}, \left(\frac{dn}{dc}\right)_{PDADMAC}}$**/ ml. g^{-1}**	$\boldsymbol{\left(\frac{dn}{dc}\right)_{IPEC}}$
0.1	0.938, 0.18700	0.062, 0.17547	0.18628
0.2	0.883, 0.18700	0.117, 0.17547	0.18565
0.3	0.834, 0.18700	0.166, 0.17547	0.18509
0.4	0.790, 0.18700	0.210, 0.17547	0.18458
0.5	0.751, 0.18700	0.249, 0.17547	0.18413
0.6	0.715, 0.18700	0.285, 0.17547	0.18372
0.7	0.683, 0.18700	0.317, 0.17547	0.18334
0.8	0.653, 0.18700	0.347, 0.17547	0.18300
0.9	0.626, 0.18700	0.374, 0.17547	0.18269
1.0	0.601, 0.18700	0.399, 0.17547	0.18240

Here, Φ_{NaCMC} and $\Phi_{PDADMAC}$ are mass fraction of NaCMC and PDADMAC, respectively

Dynamic light scattering (DLS) measurements:

We first performed the CONTIN analysis on the derived electric field autocorrelation (through Siegert's relationship) to identify the probable sizes present without assuming a predefined number of components. The fitted electric field autocorrelation function is shown in Fig. SI-4. The python program used for the CONTIN analysis is available at https://github.com/antgi1/continizer.The amplitude-weighted size distribution, obtained from the CONTIN analysis, reveals the presence of two distinct particle populations in most of the cases, as depicted in the inset of Fig. SI-4 and therefore, we decided to analyze the intensity autocorrelation function using a bimodal exponential function. The model fitting the intensity autocorrelation function is shown in Fig.SI-5.

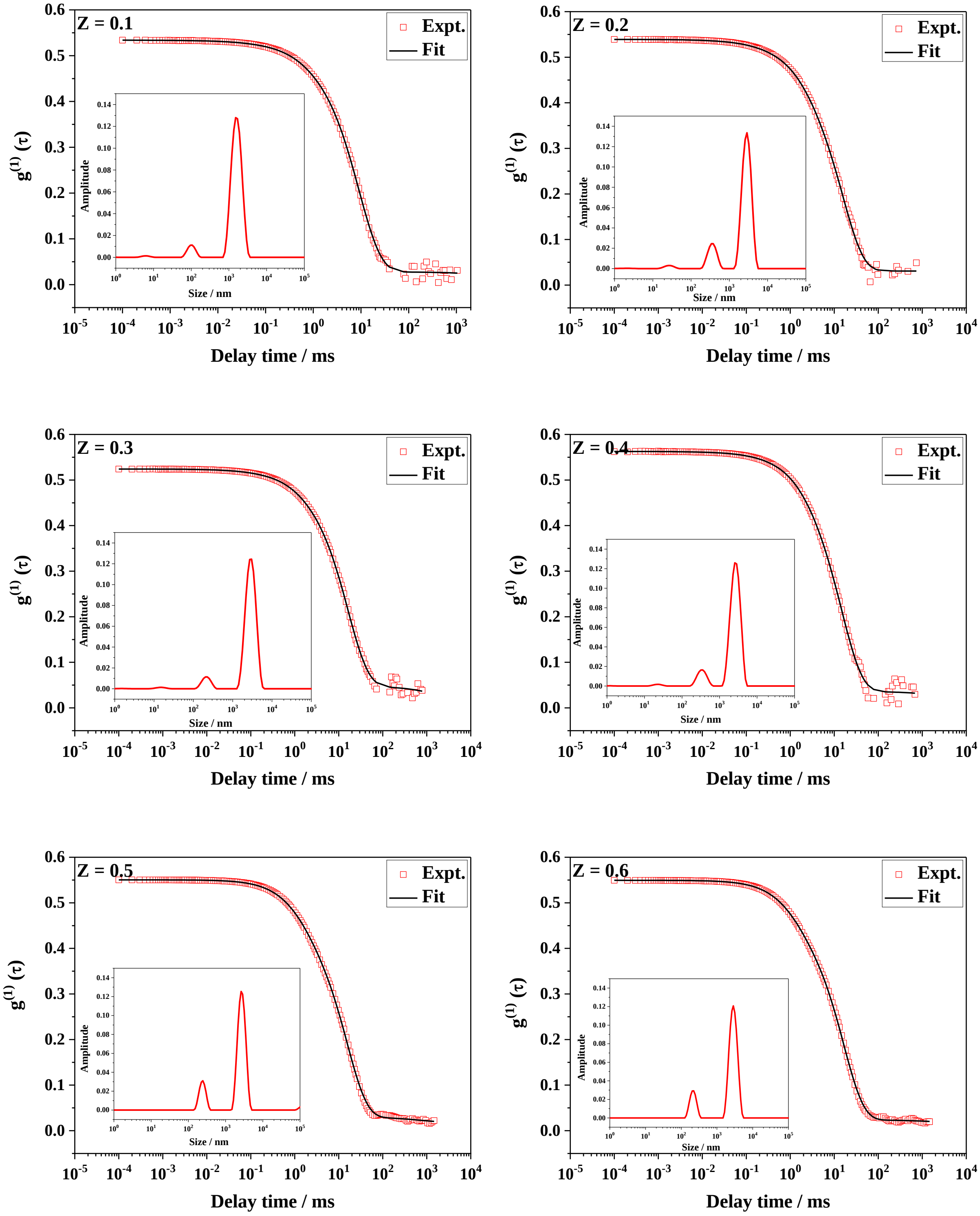
Z = 0.1
Z = 0.2
Z = 0.3
Z = 0.4
Z = 0.5
Z = 0.6
Expt.
Fit
g(1) (τ)
Delay time / ms
Amplitude
Size / nm

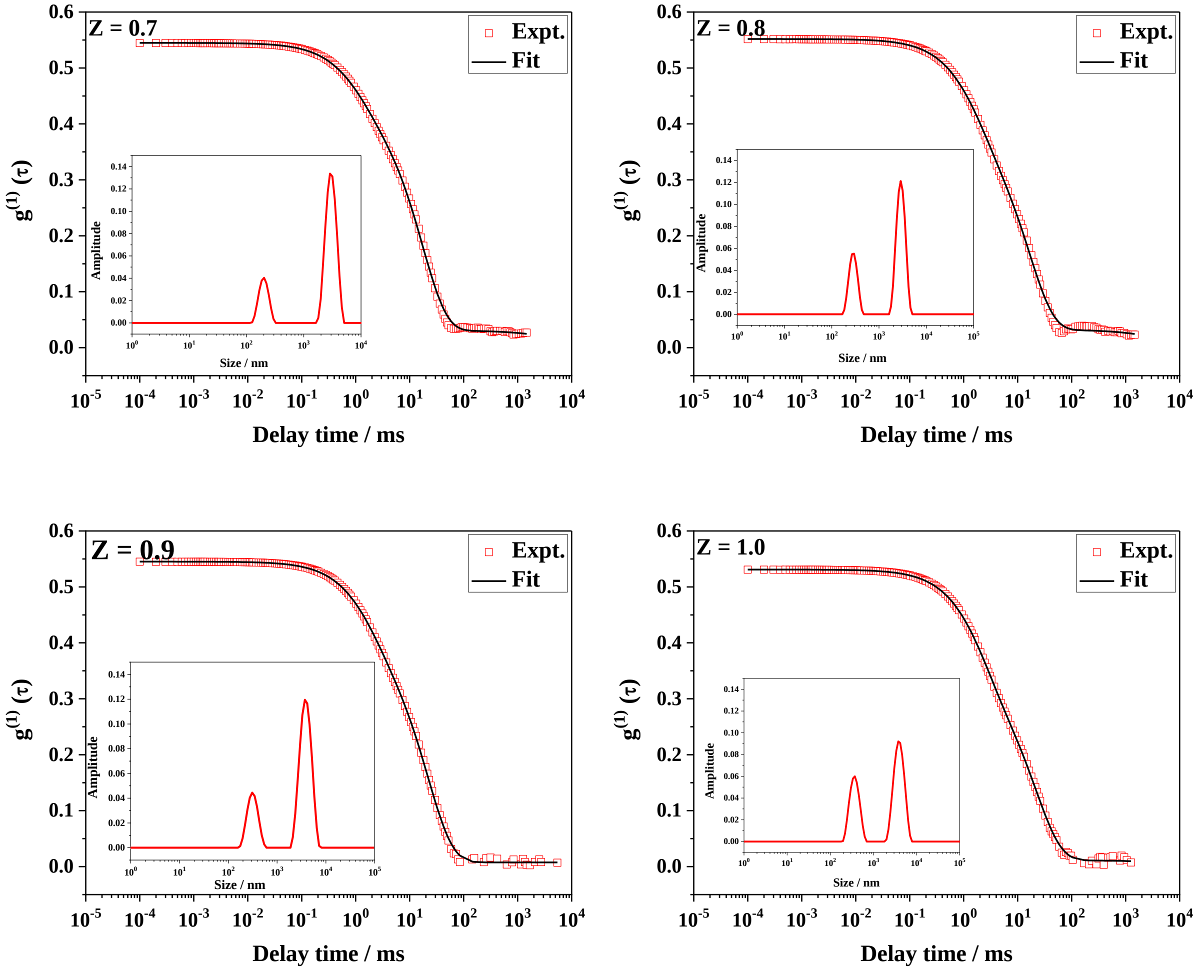

Fig. SI-4: *The electric-field autocorrelation functions at different charge ratios. The solid line represents the CONTIN fit. Inset figure shows the normalized amplitude-weighted size distribution of the IPECs obtained from CONTIN analysis*.

The relative amplitude values for each size distribution

Charge Ratio (Z)	Contribution of peaks	Amplitude at maximum peak height	Total amplitude (area under the peak)
0.1	1st peak (~5.96 nm)	0.0013	0.007
	2nd peak (~106 nm)	0.011	0.064
	3rd peak (~1511 nm)	0.128	0.929
0.2	1st peak (~25.5 nm)	0.003	0.022
	2nd peak (~339 nm)	0.024	0.148
	3rd peak (~2888 nm)	0.134	0.830
0.3	1st peak (~204 nm)	0.011	0.117
	2nd peak (~3023 nm)	0.124	0.883
0.4	1st peak (~319 nm)	0.016	0.122
	2nd peak (~2550 nm)	0.126	0.878
0.5	1st peak (~245 nm)	0.031	0.187
	2nd peak (~2618 nm)	0.125	0.813
0.6	1st peak (~223 nm)	0.029	0.171
	2nd peak (~2871 nm)	0.121	0.829
0.7	1st peak (~203 nm)	0.040	0.208
	2nd peak (~2871 nm)	0.134	0.792
0.8	1st peak (~296 nm)	0.055	0.303
	2nd peak (~2871 nm)	0.121	0.697
0.9	1st peak (~309 nm)	0.044	0.252
	2nd peak (~3698 nm)	0.119	0.748
1.0	1st peak (~372 nm)	0.059	0.374
	2nd peak (~3766 nm)	0.092	0.626

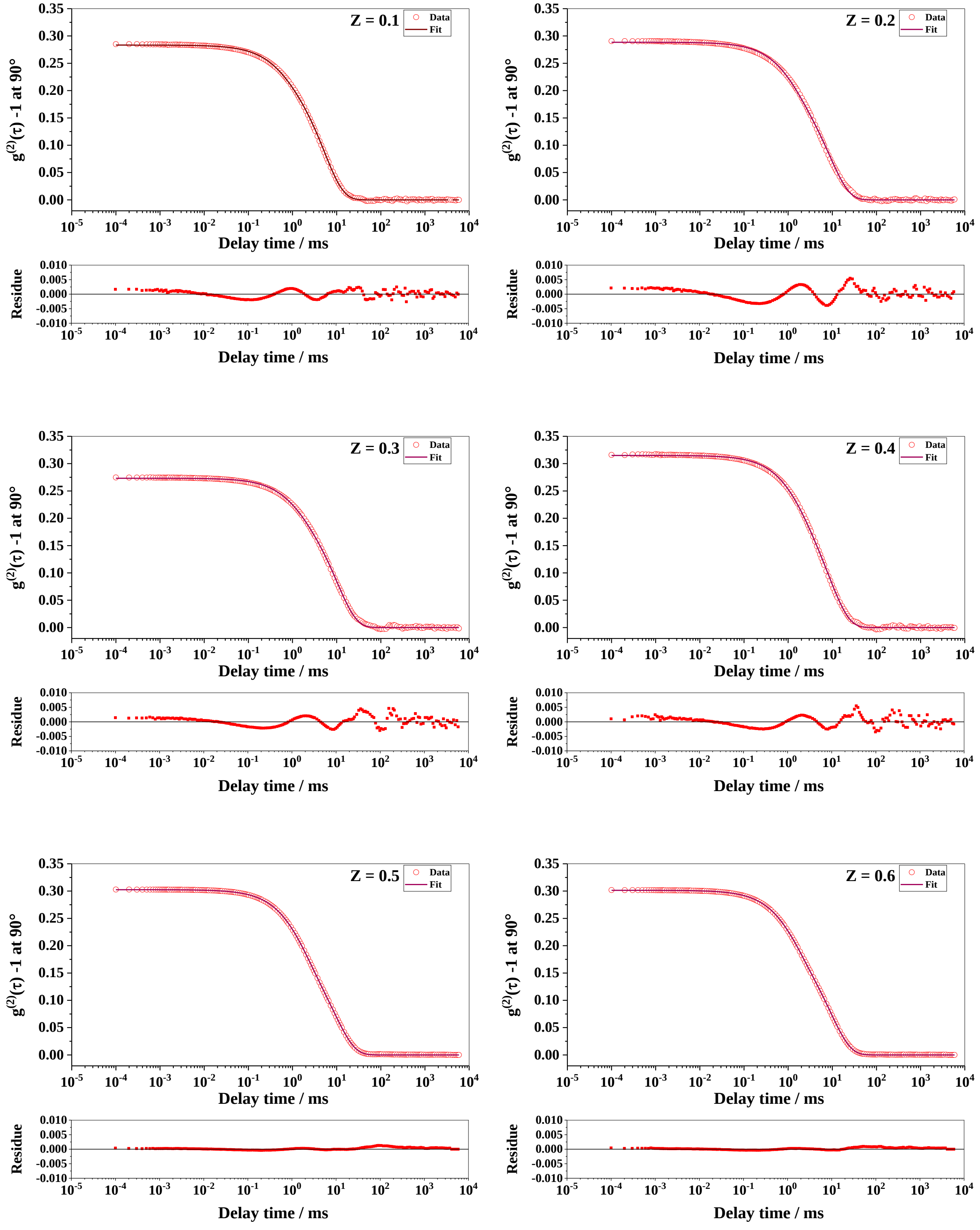
Z = 0.1
Z = 0.2
Z = 0.3
Z = 0.4
Z = 0.5
Z = 0.6
Data
Fit
g(2)(τ) -1 at 90°
Delay time / ms
Residue

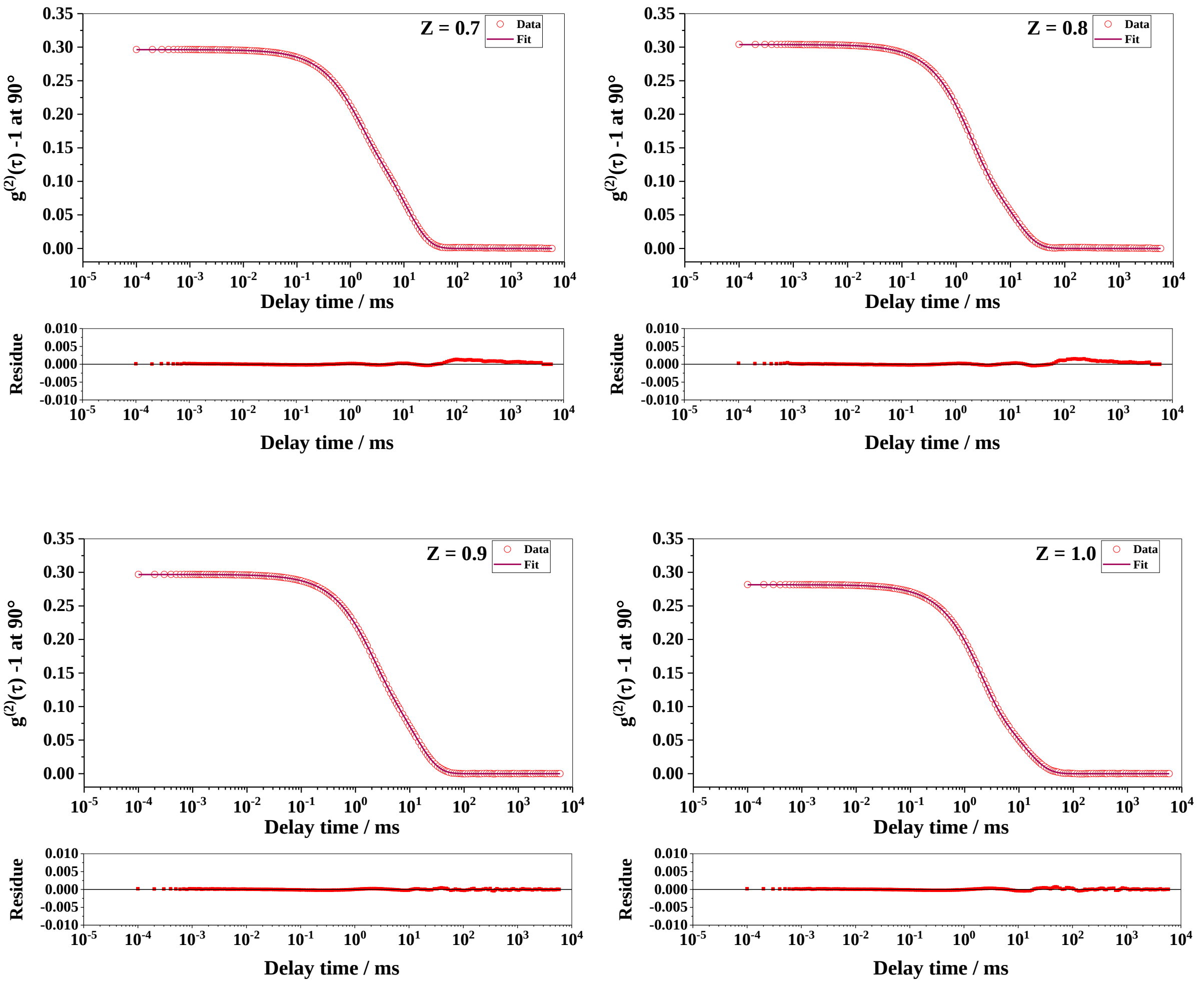

Fig. SI-5: *The DLS correlogram with the bimodal exponential fitting depicted as solid lines. The bottom panel shows the variation in residue with respect to the model fitting.*

S4. Estimate of the average spacing of the polyelectrolyte chains:

If one assumes the polyelectrolyte chains in solution to be arranged on the edges of space-filling cubes then the average spacing (d) between the chains would be given as:

$$\mathrm{d} = \sqrt{\frac{3 \cdot (m_p/d_p)}{\phi \cdot \rho_p}}$$

where m_p/d_p is the mass per length of the polymer chain, Φ the volume fraction of the polymer and ρ_p its density. For the NaCMC employed we may assume a density of 1.5 g/mL (resulting in a volume fraction of 0.0033), an effective molecular weight of 225.7 g/mol and a length of a monomer unit of 0.5 nm. With these parameters we arrive at a value of 21.3 nm, which is only somewhat smaller than the actually observed experimental value, thereby confirming that the NaCMC chains are rather stretched in solution.

S5. Variation of absolute SANS intensity at different Q values:

SANS profiles show a systematic increase in intensity value at Q = 0.04-0.2 nm^{-1} with increasing Z. The variation in intensity value at Q=0.1 nm^{-1} is shown in Fig. SI-4. In contrast to that, at lower range of Q = 0.01-0.04 nm^{-1}, one observes the highest intensity at Z = 0.2, followed by a systematic decrease to the lowest value at Z = 0.6, and subsequently it increases again with further increase of Z. Fig. SI-6 shows the variation in absolute intensity at Q = 0.01 nm^{-1}. It is noteworthy that at Z>0.6, samples are unstable and phase separation happened during SANS measurements. Thus, the variation in intensity at higher Z is also attributed to the phase stability of the samples. In addition to that, intensity value remained nearly constant with variation in Z at Q = 0.05 nm^{-1} as depicted in Fig. SI-4. This iso-intensity cross-over indicates the formation of local globular domains which are embedded in a larger network structure and becomes more pronounced with increasing Z.

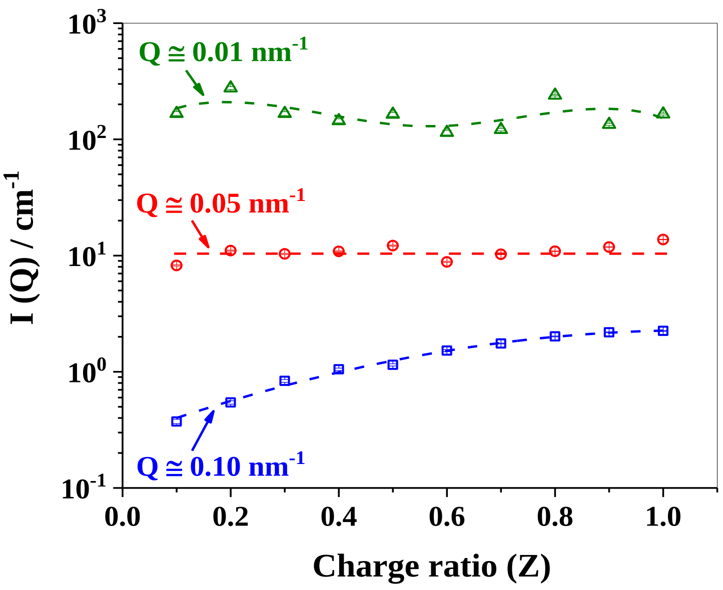

***Fig. SI-6**: The absolute intensity variation as a function of the charge ratio at different Q values. The dotted lines denote 'guide-to-eye'.*

S6. Estimation of peak position (Q^*) of scattering profiles in Kratky scale:

The bell-shaped Kratky profiles were fitted using the following Gaussian curve around the highest intensity value. The fitted profiles and the variation in Q^* are shown in Figure SI-5.

$$y(Q) = y_0 + y_1 * e^{-\frac{(Q-Q^*)^2}{2\sigma^2}}$$

Here 'y_0' is the intensity baseline and 'y_1' denotes a scaling constant of the Gaussian profile. 'Q^*' and 'σ' stand for the mean and width of the Gaussian distribution, respectively. The increase in Q^* with increasing charge ratio (Z) suggests reduction in aggregate size.

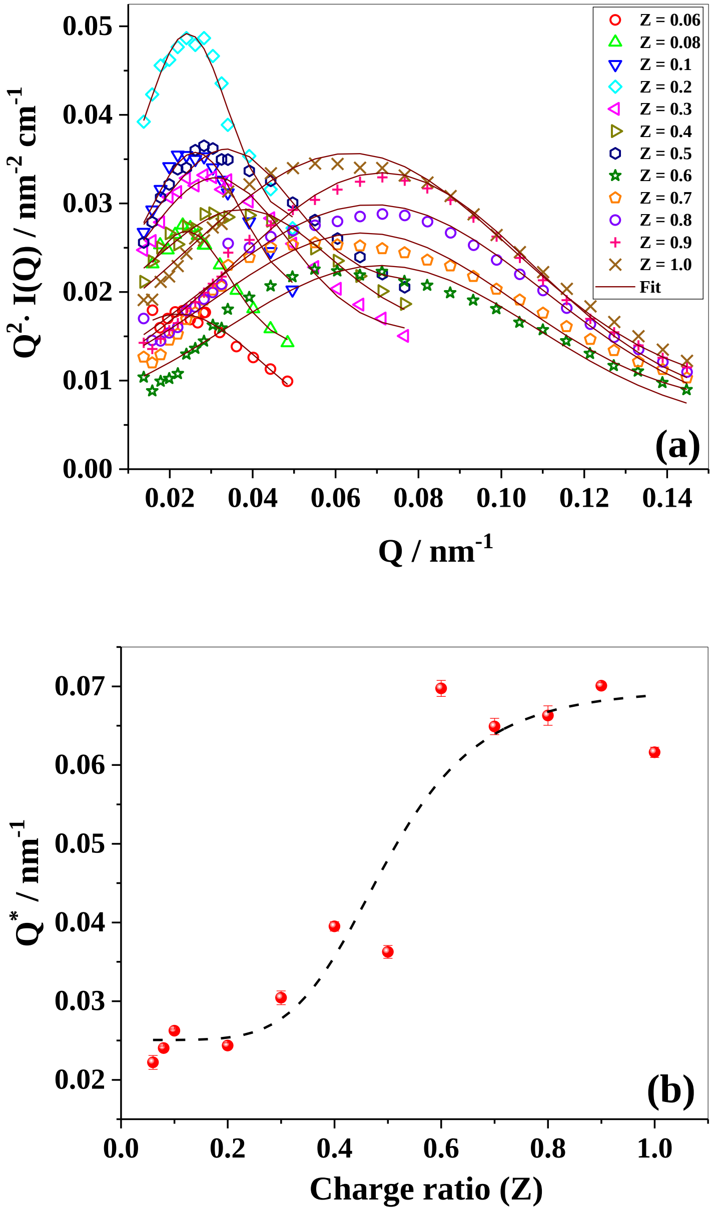

Fig. SI-7: *(a) Depicts the fitting of Gaussian distribution to the Kratky profiles. (b) The variation in peak position (Q^*) of scattering profiles in Kratky scale obtained by fitting a Gaussian profile near the peak. Dashed line denotes a 'guide-to-eye'.*

S7. Inverse Fourier transform (IFT) analysis of SANS data:

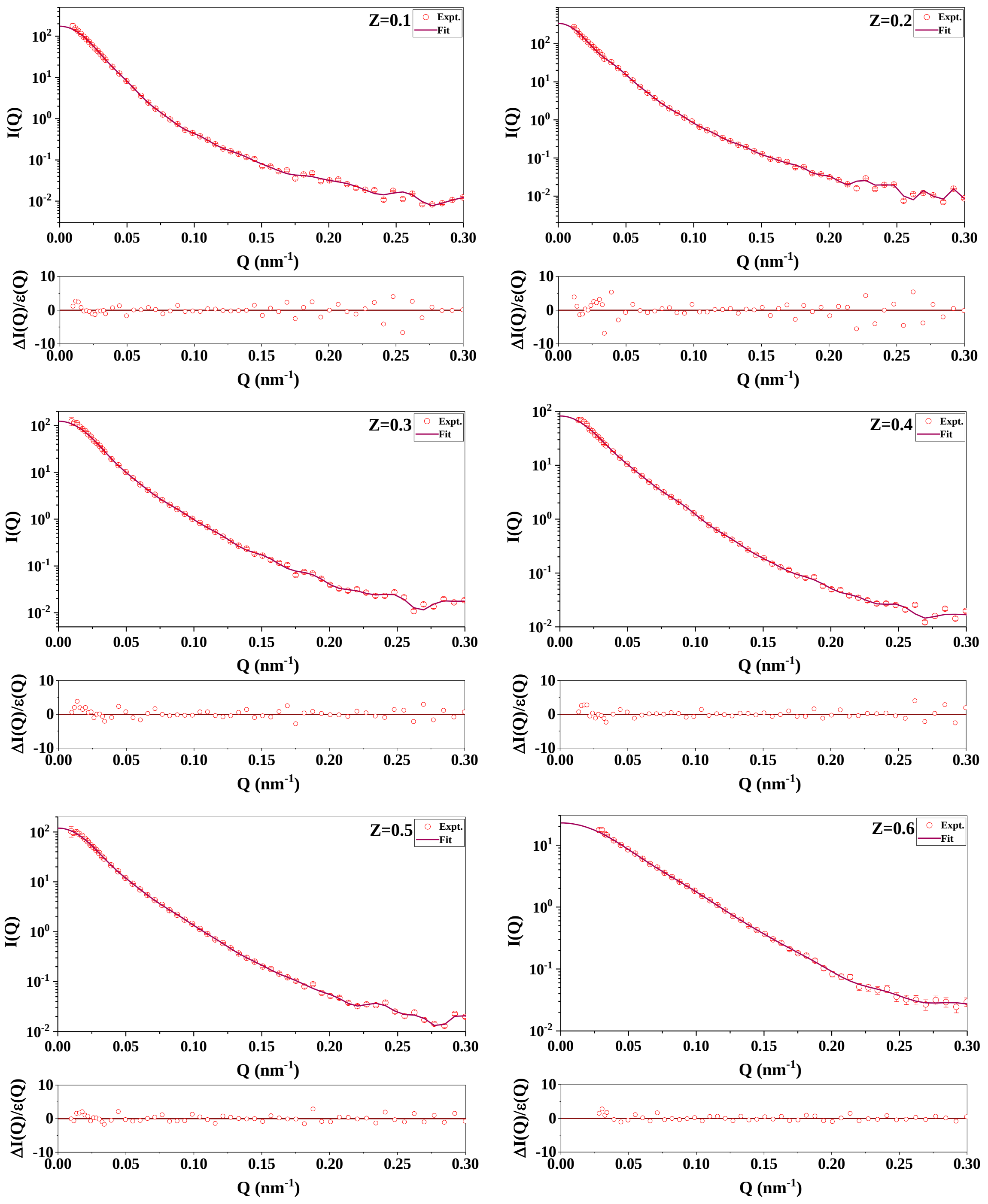

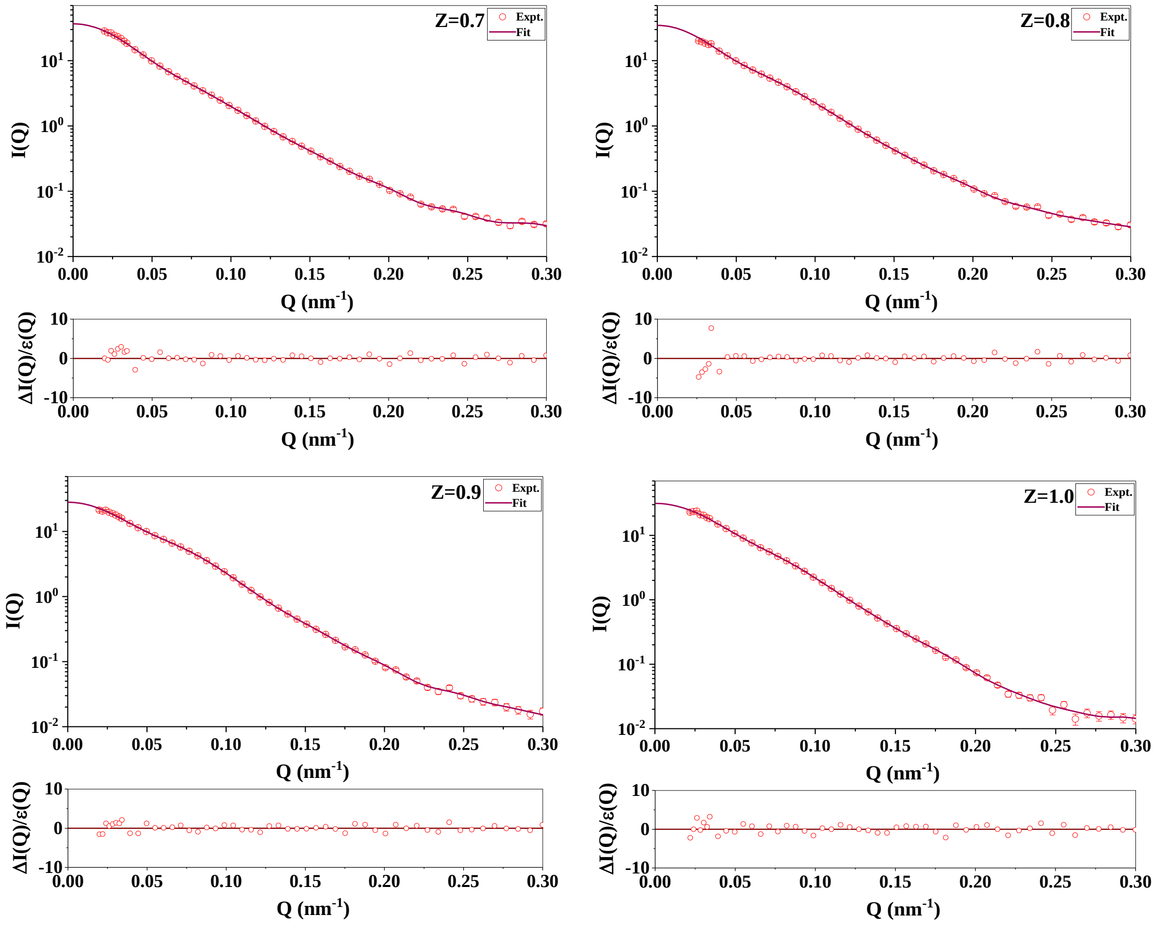

Fig. SI*-8: (Top panel) PDDF fitting to the experimental profiles by inverse Fourier transform analysis. (Bottom panel) Residue with respect to the fitting.*

S8. Ab-initio modelling of SANS data:

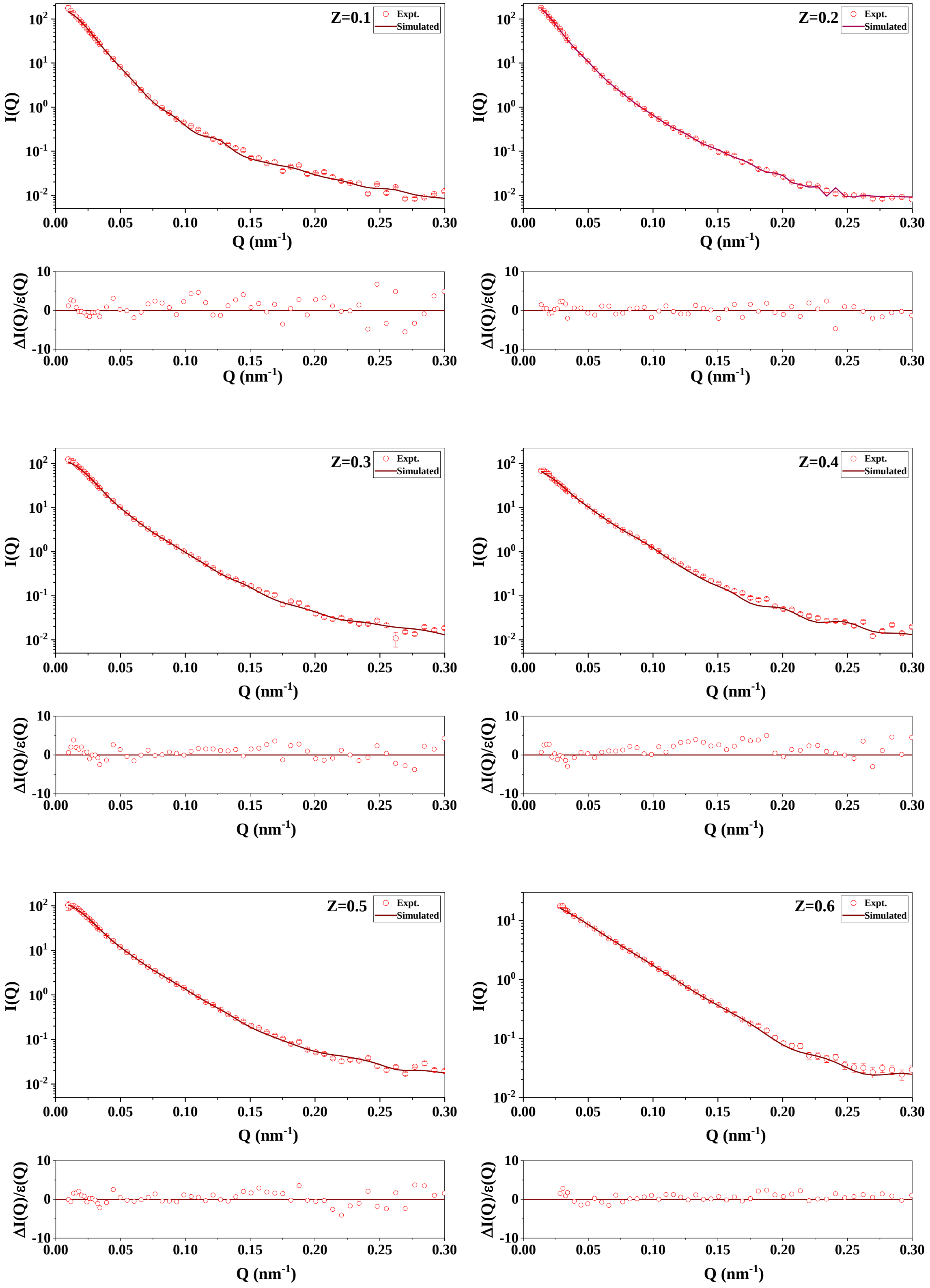

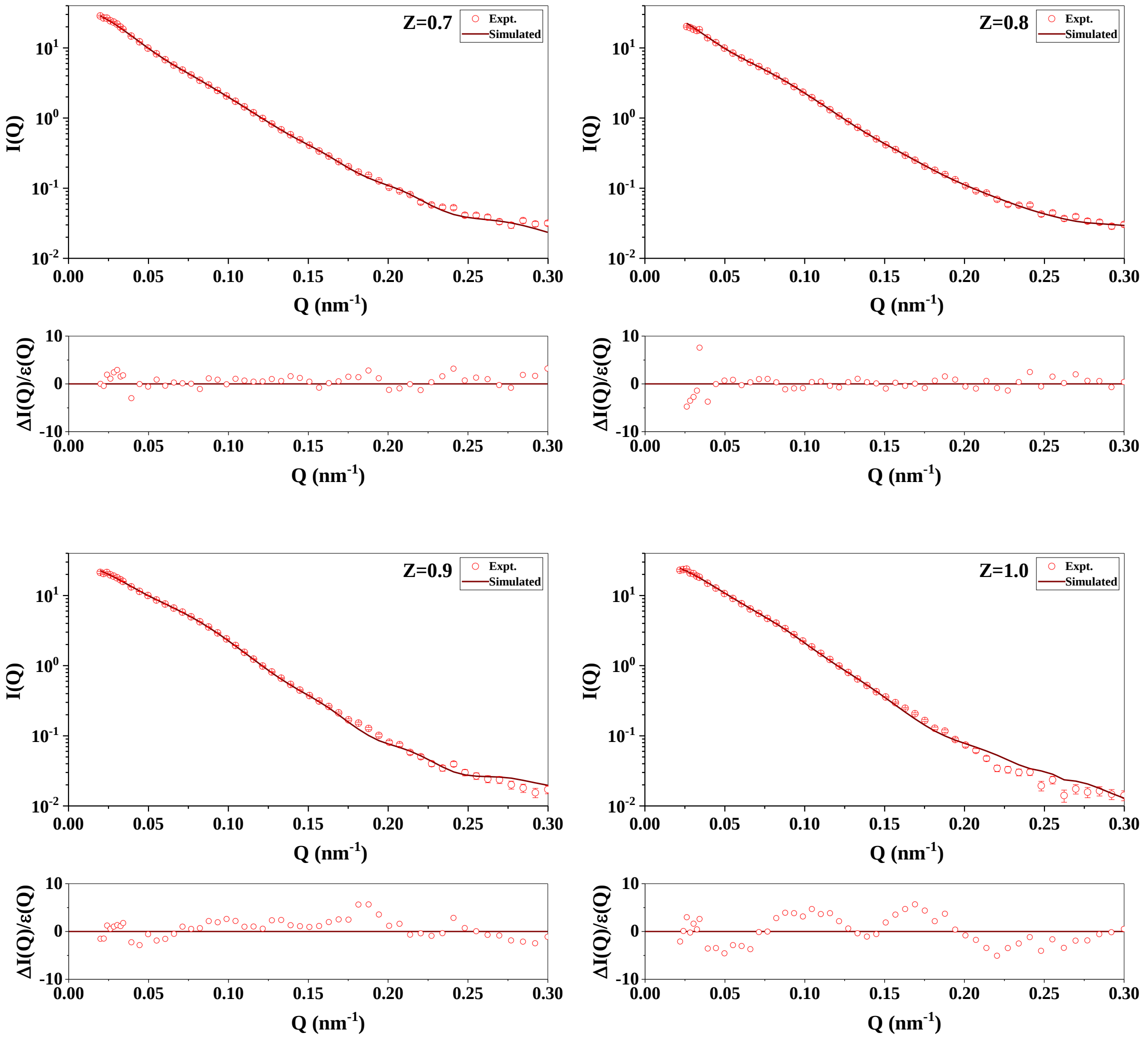

Fig. SI-9*: Ab initio model fitting obtained from DAMMIN to the experimental SANS profiles (Top panel). Solid lines represent the simulated curve. The bottom panels show the residue with respect to the model fitting.*

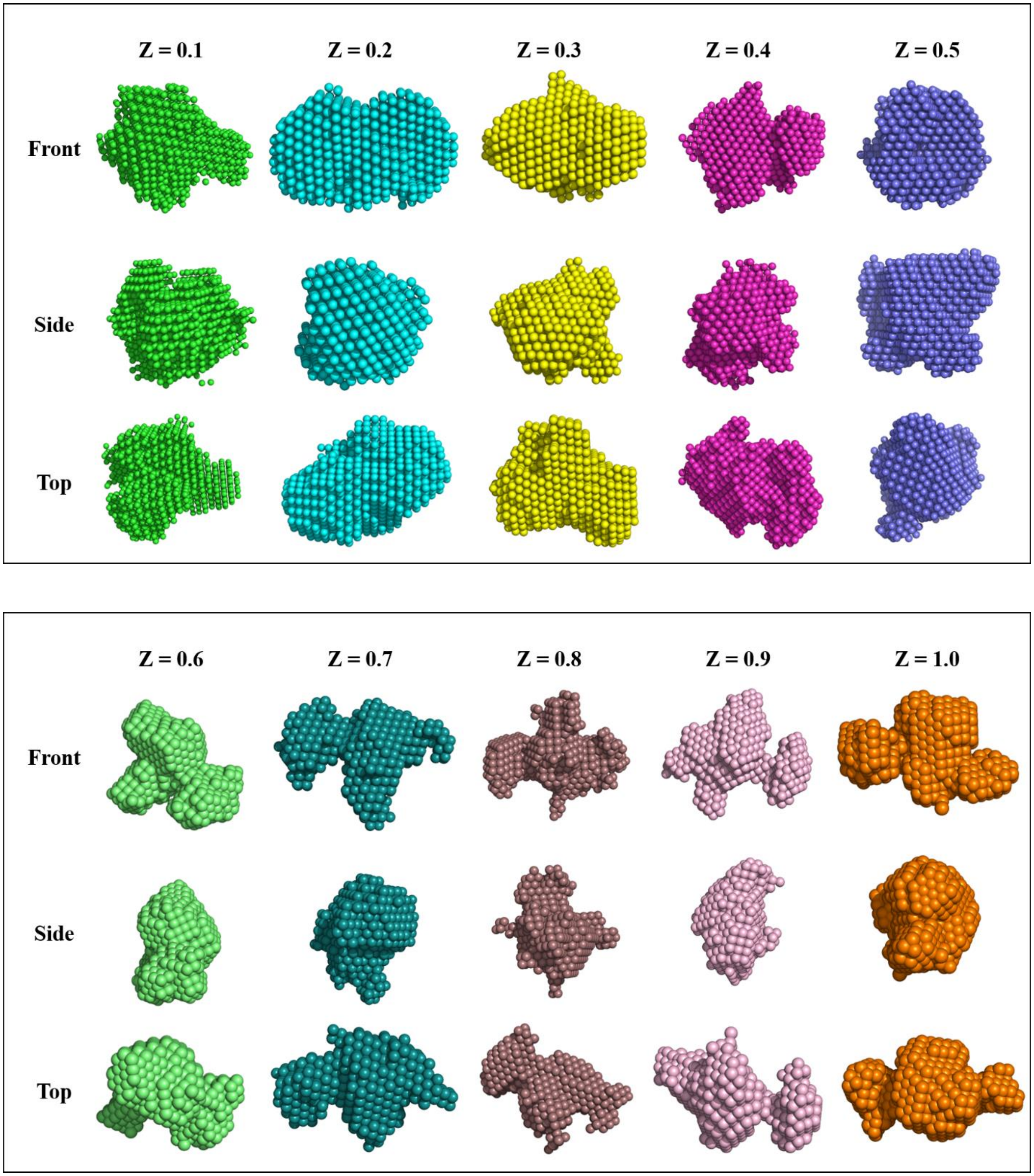

Fig. SI-10*: Orthogonal projections of the constructed ab initio model of IPECs at different charge ratios.*

S9. Calculation of solvation water:

Neutron scattering length density (SLD) of anhydrous IPECs (η_{anhyd}) was calculated as follow:

$$\eta_{anhyd} = \phi_+\eta_+ + \phi_-\eta_-$$

where '$\phi_{+/-}$' are solute volume fraction of polycations and polyanions, respectively as per the sample preparation protocol. '$\eta_{+/-}$' are neutron SLDs of polycations and polyanions known from their chemical composition. The density of PDADMAC and NA CMC was considered to be ~1.04 g/cc and ~1.6 g/cc, respectively. Now, if we assume that finite amount of water penetrates into the formed macromolecular complex, then the effective neutron SLD (η_{IPEC}) of such hydrated complex can be written as:

$$\eta_{IPEC} = \phi_w\eta_w + (1 - \phi_w)\eta_{anhyd}$$

where 'ϕ_w' denotes the volume fraction of water inside the complex, and 'η_w' is the neutron SLD of solvent water (D_2O). Experimentally one can determine the contrast $[(\Delta\eta)^2]$ of the aggregates and that can be used to determine the volume fraction of water present inside the aggregates as follows:

$$\Delta\eta = \eta_w - \eta_{IPEC}$$

$$\text{or, } \eta_{IPEC} = \eta_w - \Delta\eta$$

$$\text{or, } \phi_w\eta_w + (1 - \phi_w)\eta_{anhyd} = \Delta\eta - \eta_w \quad \text{(using previous equation)}$$

$$\text{or, } \phi_w(\eta_w - \eta_{anhyd}) = (\eta_w - \eta_{anhyd}) - \Delta\eta$$

$$\text{or, } \phi_w = 1 - \frac{\Delta\eta}{(\eta_w - \eta_{anhyd})}$$

References:

(1) Provencher, S.W. A constrained regularization method for inverting data represented by linear algebraic or integral equations *Comput. Phys. Commun*., **1982**, 27, 213-227. https://doi.org/10.1016/0010-4655(82)90173-4
